# The Electrodeposition of Silver from Supercritical Carbon Dioxide/Acetonitrile

**DOI:** 10.1002/celc.201300131

**Published:** 2013-11-26

**Authors:** Philip N Bartlett, Magdalena Perdjon-Abel, David Cook, Gillian Reid, William Levason, Fei Cheng, Wenjian Zhang, Michael W George, Jie Ke, Richard Beanland, Jeremy Sloan

**Affiliations:** [a] Chemistry, University of Southampton, Southampton SO17 1BJ (United Kingdom) E-mail: pnb@soton.ac.uk; [b] School of Chemistry, University of Nottingham University Park, Nottingham, NG7 2RD (United Kingdom); [c] Department of Physics, University of Warwick Gibbet Hill Road, Coventry, CV4 7AL (United Kingdom)

**Keywords:** cyclic voltammetry, electrochemistry, silver, supercritical fluid, voltammetry

## Abstract

Cyclic voltammetry of silver coordination complexes in acetonitrile and in a single-phase supercritical carbon dioxide/acetonitrile (scCO_2_/CH_3_CN) system is reported. Five silver precursors are investigated: (1,5-cyclooctadiene)(hexafluoroacetylacetonato) silver(I) [Ag(hfac)(COD)], (hexafluoroacetylacetonato)(triphenylphosphine) silver(I) [Ag(hfac)(PPh_3_)], (perfluorooctanoato)bis(triphenylphosphine) silver(I) [Ag(CF_3_(CF_2_)_6_CO_2_)(PPh_3_)_2_], tetrakis(triphenylphosphine) silver(I) tetrafluoroborate [Ag(PPh_3_)_4_][BF_4_] and tetrakis(acetonitrile) silver(I) tetrafluoroborate [Ag(CH_3_CN)_4_][BF_4_]. Of these, [Ag(CH_3_CN)_4_][BF_4_] is found to be the most suitable for electrodeposition of silver from scCO_2_/CH_3_CN.

## 1. Introduction

There are relatively few accounts of silver electrochemistry in non-aqueous solutions,[1, 2] and even less in non-aqueous supercritical media.[3–5] There is an interest in the electrodeposition of silver electrical contacts from non-aqueous solvents on water-sensitive ceramic superconductors, in which the lower resistivity of silver and its better adhesion to these materials, compared to copper, is an advantage.[6] Silver may also be the next preferred ultra-large-scale integration/interconnect material, owing to its lower resistivity (reduced resistance–capacitance delay times) and higher electro-migration resistance than copper.[7–9] Ionic liquids have been used to produce silver thin films, nanoparticles and nanowires.[10, 11] A comparison of non-aqueous solvents, provided by Titova et al.,[2] showed that the overpotential for silver reduction was the lowest in acetonitrile because of its low donor number.

The application of non-aqueous supercritical fluids (SCFs) for metal electrodeposition is a valuable method for filling confined spaces, which is becoming a more significant matter as miniaturisation of devices progresses.[12] A review on the electrochemical experiments at high pressures and temperatures is provided in the literature.[13] The level of difficulty of performing such experiments is also described therein.

SCFs are dense gases that exist above the critical temperature and pressure of a pure substance. They combine the high diffusivity of gases with the solvating properties of liquids.[14, 15] Supercritical carbon dioxide (scCO_2_) was chosen for this study because it is widely used in industry and research (extraction, chromatography, chemical deposition, etc.), it is also inexpensive and non-toxic. Carbon dioxide attains the supercritical state at near-ambient temperatures (*T_c_*=304.13 K) and at relatively moderate pressures (*p_c_*=73.8 bar).[16] It has a low dielectric constant, *ε* ≈ 2, which creates a technical problem in performing electrochemical experiments because it results in low salt dissociation and, hence, low ionic conductivity. There are, however, means to increase conductivity in scCO_2_, that is, the addition of a polar co-solvent, the use of a salt with a large hydrophobic cation and anion (e.g. tetraalkylammonium salts), the use of a fluorinated ion for increased solubility, or a combination of the above.[17] Acetonitrile, a polar aprotic solvent with a dielectric constant of *ε*=37.5, was employed. It was found to be more suitable than methanol in our supercritical system.[18] Approximately 15 % (v/v) of the co-solvent was added; this amount was chosen after phase-behaviour studies, which indicated a significantly enhanced solubility of the supporting electrolyte and solute compared to using approximately 13 % (v/v) acetonitrile. Tetraalkylammonium salts were employed as supporting electrolytes at a concentration of 20 mm. The choice of concentration was based on phase-diagram studies and previous work reported in the literature.[19, 20]

Herein, we report the electrodeposition of silver from scCO_2_/CH_3_CN using five different silver(I) complexes. A successful reagent for supercritical fluid electrodeposition (SCFED) must meet several criteria, that is, it must have good solubility and stability in the low polarity SCF, give clean electrodeposits at accessible potentials, and avoid undesirable electrochemistry or fouling at the counter electrode. In this study, we examined (1,5-cyclooctadiene)(hexafluoroacetylacetonato) silver(I), [Ag(hfac)(COD)], which has previously been used for thermal and chemical vapour deposition of silver films or nanoparticles, several triphenylphosphine complexes with fluorinated organic co-ligands that were expected to give good solubility in the SCF, and tetrakis(acetonitrile) silver(I) tetrafluoroborate, [Ag(CH_3_CN)_4_][BF_4_], in which the ligands were the same as the co-solvent and, thus, avoided introducing any other species into the system upon ligand release during electrodeposition. We describe the voltammetry of the complexes in liquid acetonitrile, followed by similar data in scCO_2_/CH_3_CN. Based upon these studies, the most suitable complex was selected and used to deposit silver both onto macroelectrodes and into anodic alumina templates to form silver nanowires.

## Experimental Section

### Reagents

Acetonitrile (Chromasolv LC-MS 99.9 %, Riedel de Haën), tetrabutylammonium tetrafluoroborate [*n*Bu_4_N][BF_4_] (Aldrich), (hexafluoroacetylacetonate) (1,5-cyclooctadiene) silver(I) [Ag(hfac)(COD)] (Aldrich), and tetrakis(acetonitrile) silver(I) tetrafluoroborate [Ag(CH_3_CN)_4_][BF_4_] (ReagentPlus 99 %, Sigma–Aldrich) were used as received. Tetrabutylammonium tetrakis[3,5-bis(trifluoromethyl)phenyl]borate [*n*Bu_4_N][B{C_6_H_3_(CF_3_)_2_}_4_] was made as described previously.[21] Tetrakis(triphenylphosphine) silver(I) tetrafluoroborate [Ag(PPh_3_)_4_][BF_4_],[22] (hexafluoroacetylacetonato)(triphenylphosphine) silver(I) [Ag(hfac)(PPh_3_)],[23] and (perfluorooctanoato)bis(triphenylphosphine) silver(I) [Ag(CF_3_(CF_2_)_6_CO_2_)(PPh_3_)_2_][24] were made by following literature methods and the purity was checked by using microanalysis and assessing appropriate spectroscopic data before use. The silver complexes were wrapped with aluminium foil and stored in the dark.

### Electrochemical Measurements

The detailed description of the set-up and apparatus for SCFED is provided in previous publications.[12, 21]The phase behaviour of the supercritical solutions was studied by using a variable volume view-cell, which has also been described in the literature.[25] The phase diagrams indicated the pressure and temperature regimes required for a homogenous solution at a chosen CO_2_, acetonitrile, supporting electrolyte and precursor composition.

The experiments were carried out in a three-electrode system. The silver-complex solutions, in acetonitrile with the supporting electrolyte, were bubbled with argon saturated with acetonitrile prior to the experiments in acetonitrile and prior to inserting the solution into the high-pressure cell for experiments in scCO_2_/CH_3_CN.

The Pt microelectrodes used in acetonitrile were fabricated in accordance with a method described in the literature.[26] The macroelectrode consisted of a 0.5 mm-diameter Pt disk sealed in glass. To prepare the microelectrodes used in the scCO_2_/CH_3_CN system, the Pt microwire was first sealed in a soda-glass capillary, then a section of it was contacted to a 0.5 mm-diameter Cu (or Pt) wire and sealed with bisphenol A epoxy resin (EpoFix, Struers) in 1.6 mm polyetheretherketone (PEEK) tubing. The microelectrodes were polished with alumina paste (1, 0.3, and 0.05 μm) on a polishing cloth (Buehler). The pseudo-reference electrodes were 0.5 mm-diameter Ag and Pt wires. The counter electrode in acetonitrile was a Pt gauze that was cleaned in flame, and in the scCO_2_/CH_3_CN system the counter electrode was either a Pt or sacrificial Ag coil.

The high-pressure cell was of a constant volume and, once it was sealed, the properties of the SCF could be isochorically changed by adjusting the temperature. The change in pressure followed that expected from the basic equations of state. This allowed the collection of data from a solution with a constant composition at varied *T* and *p*.

### Templates

Two sources of anodic alumina membranes were used as templates for electrodeposition: 1) Whatman anodised aluminium oxide (AAO) membranes with, nominally, 20 and 200 nm pore diameters, and 2) Synkera AAO membranes with, nominally, 13 nm diameter pores. Both types are 60 μm thick and supplied in small sections (typically 13 mm diameter disks or 1 cm squares). The Sykera membranes are mechanically more robust with a more-consistent pore diameter throughout [as assessed by scanning electron microscopy (SEM) and transmission electron microscopy (TEM) of cross-sections].

The membranes were prepared as electrodes as follows. One face of the Synkera membrane was coated with an evaporated film of Cr (5 nm) for adhesion, followed by 100 nm of gold. Small sections of the coated membrane (0.2–1 cm^2^) were contacted to steel wires of 0.5 mm diameter by using silver-loaded epoxy resin (ITW Chemtronics CW2400). The wire itself was insulated with a length of PEEK tubing (1.6 mm outer diameter and 0.5 mm inner diameter), similar to the electrodes described above. The silver epoxy contact, gold-coated face of the membrane, and any exposed steel were then insulated with epoxy resin (EpoFix, Struers) such that the only remaining gold that was accessible to the plating solutions was at the bottom of the alumina pores. The Whatman membranes were made conductive by sputtering 200–400 nm of gold on to one face. Contacts were then made to Ag or Pt wires (sealed in PEEK as described above) by using silver epoxy or, in some cases, Cu or C adhesive tape. Contacts and any exposed gold were insulated by using the same epoxy resin described above.

Images of deposited wires and membranes were obtained by using a JSM-6500F field-emission gun SEM (JEOL). For SEM, prior to imaging, the alumina membranes were sputtered with gold for 45 s (ca. 15 nm layer) to enhance the conductivity of the substrate and obtain images of better quality. For images of the freestanding wires, the AAO was dissolved in 1 m NaOH. Cross-section TEM specimens were prepared by grinding, polishing and Ar^+^-ion milling with liquid-nitrogen cooling, and examined in a JEOL 2000FX microscope operated at 200 kV.

## 2. Results and Discussion

### 2.1. Silver Complexes

**Figure 1:**
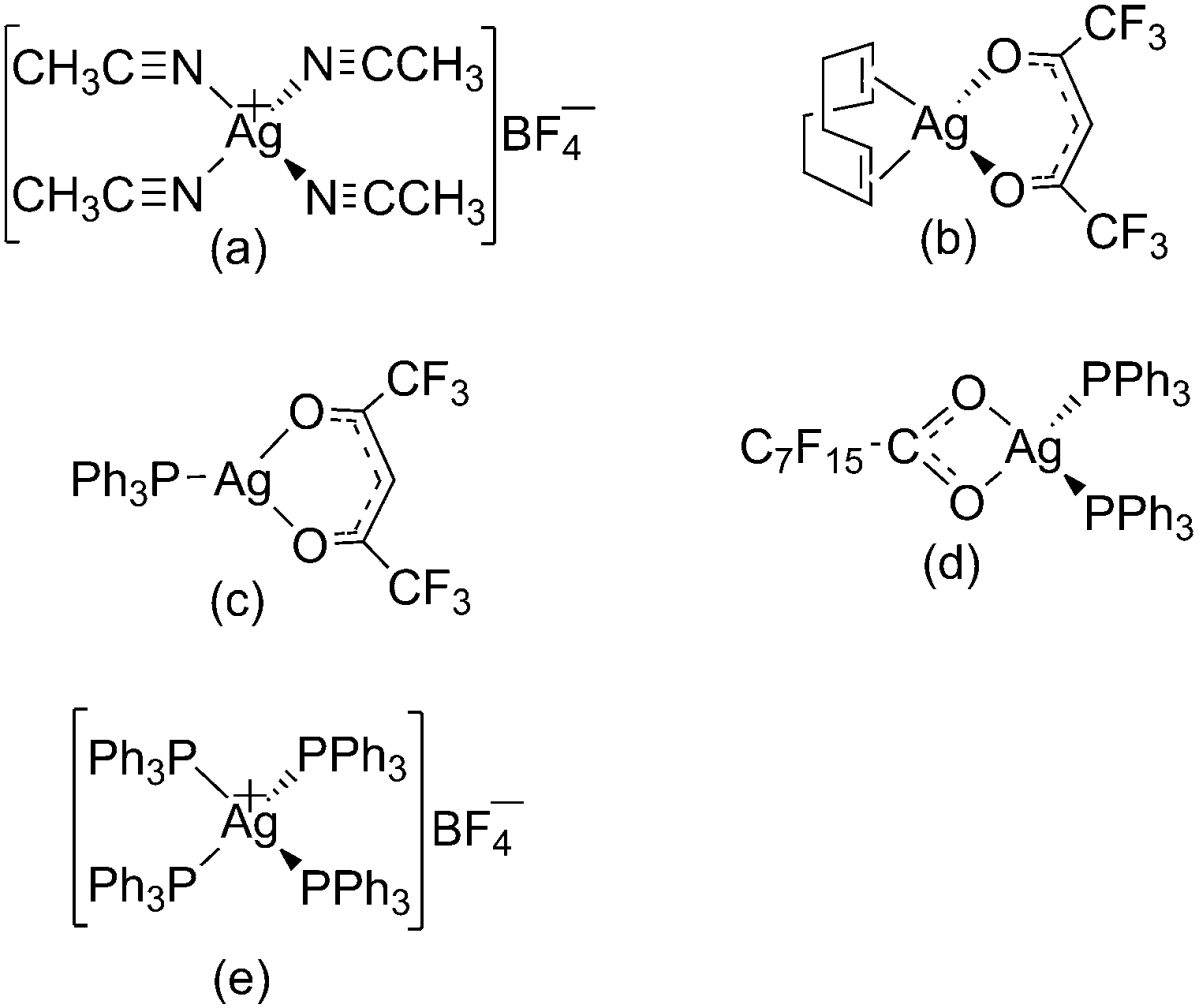
Structures of the silver precursors used: a) [Ag(CH_3_CN)_4_][BF_4_], b) [Ag(hfac)(COD)], c) [Ag(hfac)(PPh_3_)], d) [Ag(CF_3_(CF_2_)_6_CO_2_)(PPh_3_)_2_] and e) [Ag(PPh_3_)_4_][BF_4_].

The five silver precursors used, [Ag(CH_3_CN)_4_][BF_4_], [Ag(hfac)(COD)], [Ag(PPh_3_)_4_][BF_4_], [Ag(hfac)(PPh_3_)] and [Ag(CF_3_(CF_2_)_6_CO_2_)(PPh_3_)_2_] (Figure 1), were obtained commercially or made by following literature methods.[22–24]

### 2.2. Cyclic Voltammetry of Silver Precursors in Acetonitrile

There is relatively little in the literature on the electrodeposition of metals from non-aqueous solutions; an overview can be found in the review by Simka et al.[27] Consequently, as a first step in our studies, we looked at the voltammetry of the different complexes in liquid acetonitrile containing tetrabutylammonium tetrafluoroborate as a background electrolyte. This also allowed us to directly compare the voltammetry in the liquid state with that in the SCF containing around 15 % acetonitrile (v/v).

#### 2.2.1. Cyclic Voltammetry of [Ag(CH_3_CN)_4_][BF_4_] in Acetonitrile

**Figure 2:**
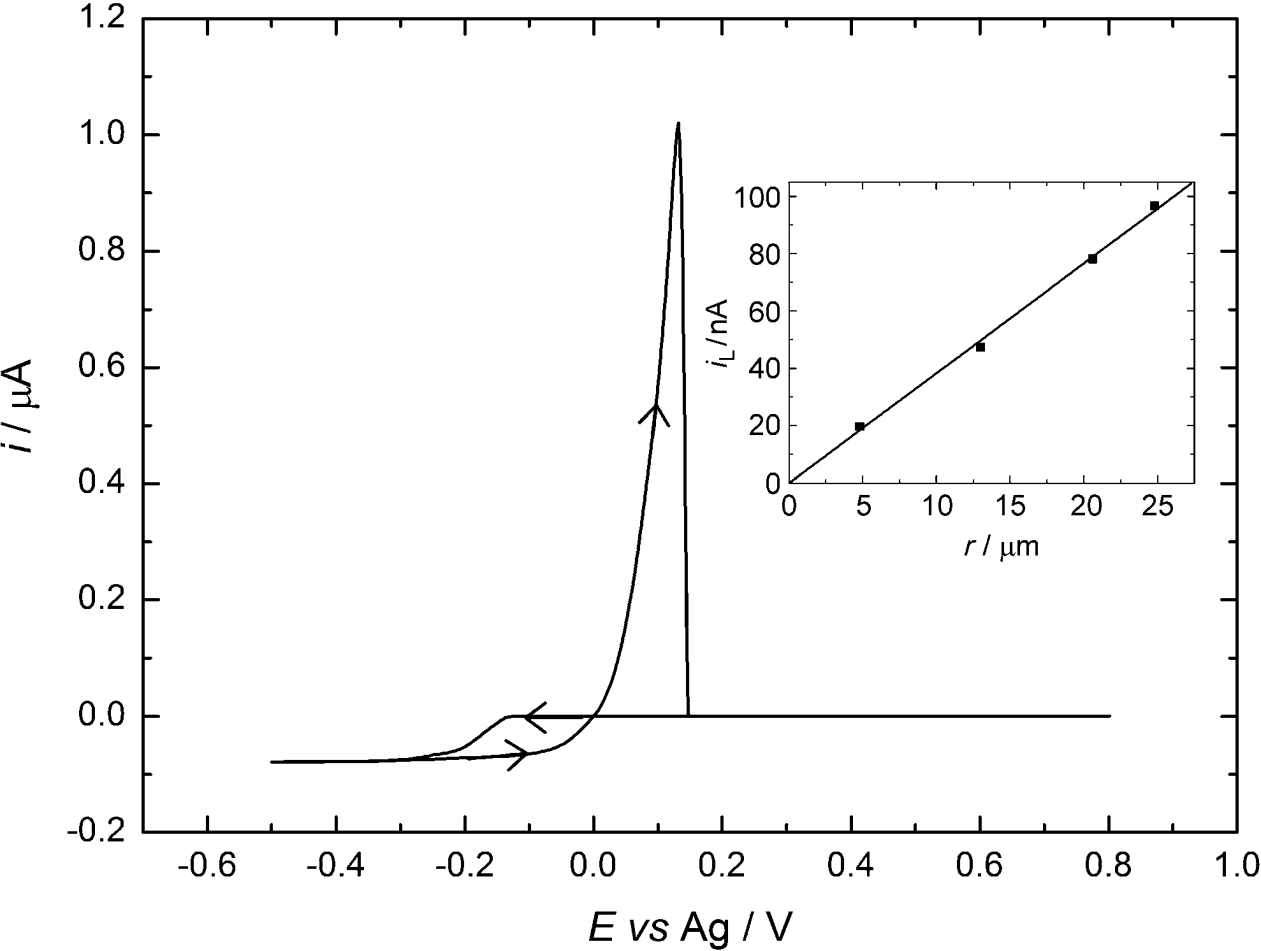
Cyclic voltammetry in 4.1 mm [Ag(CH_3_CN)_4_][BF_4_] + 20.5 mm [*n*Bu_4_N][BF_4_] in acetonitrile at a 41 μm Pt microdisk. Recorded at a scan rate of 0.05 V s^−1^ and at a temperature of 297±1 K. The inset shows the correlation between the limiting current and electrode size.

Figure 2 shows a typical cyclic voltammogram for [Ag(CH_3_CN)_4_][BF_4_] in acetonitrile. The voltammetry shows the classic behaviour expected for electrodeposition and stripping at a microdisk electrode, with a clearly defined nucleation loop on the cathodic scan and an anodic stripping peak at 0.13 V versus Ag on the return scan. The onset of Ag deposition occurs at −0.174 V versus Ag, corresponding, in this case, to a nucleation overpotential of 174 mV. On the microdisk electrode, the plating current reaches a mass-transport-limited value of 78 nA at the cathodic limit. The inset shows a plot of the limiting current for silver deposition recorded at four different microelectrodes as a function of the microelectrode radius. From the slope and by using the expression for the limiting current at a microdisk electrode [Eq. (1)]:


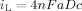
(1)

where *i*_L_ is the mass-transport-limited current, *D* is the diffusion coefficient, *a* is the electrode radius and *c* is the bulk concentration, we obtain an estimate of the diffusion coefficient for [Ag(CH_3_CN)_4_]^+^ at 297±1 K of (2.42±0.22)×10^−5^ cm^2^ s^−1^.

The data from this and other voltammograms recorded for different concentration of [Ag(CH_3_CN)_4_][BF_4_] are given in the Supporting Information. In all cases, the voltammetry was well-behaved with a clear nucleation loop and stripping peak for the deposited silver. The average Faradaic efficiency for the deposition (defined as the ratio of the deposition charge to the stripping charge) was 0.97 and the nucleation overpotential varied between 57 and 175 mV for the different electrodes (see Table S1 in the Supporting Information).

The experiments in SCF were carried out at 308 K. We, therefore, recorded voltammetry for 2.5 mm [Ag(CH_3_CN)_4_][BF_4_] in acetonitrile at 308 K for direct comparison. At 308 K, the limiting current is approximately 17.5 % higher, corresponding to a diffusion coefficient of (3.02±0.30)×10^−5^ cm^2^ s^−1^. At 308 K, the Faradaic efficiency remains high (0.94) and there is a decrease in the nucleation overpotential (see Table S1 in the Supporting Information).

#### 2.2.2. Cyclic Voltammetry of [Ag(hfac)(COD)] in Acetonitrile

**Figure 3:**
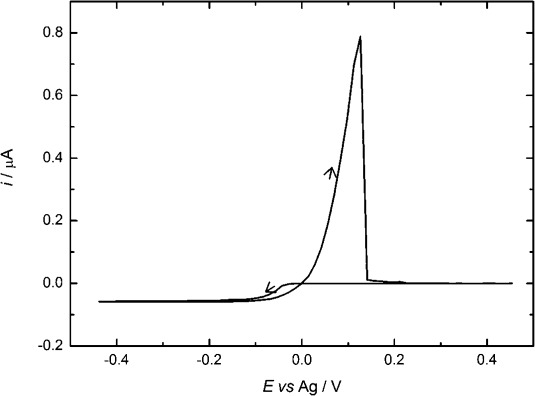
Cyclic voltammogram in 5.5 mm [Ag(hfac)(COD)] + 21.5 mm [*n*Bu_4_N][BF_4_] in acetonitrile for a 25 μm Pt electrode. Recorded at a scan rate of 0.05 V s^−1^ and at a temperature of 297±1 K.

Figure 3 shows a typical microelectrode cyclic voltammogram for [Ag(hfac)(COD)] in acetonitrile with [*n*Bu_4_N][BF_4_] as the supporting electrolyte; again we see the typical features for Ag deposition and stripping. The estimated diffusion coefficient, calculated from several separate experiments at 297±1 K was (2.2±0.2)×10^−5^ cm^2^ s^−1^. The Faradaic efficiency for the deposition was 0.99 and the nucleation overpotential typically 59 mV.

#### 2.2.3. Cyclic Voltammetry of [Ag(hfac)(PPh_3_)] in Acetonitrile

**Figure 4:**
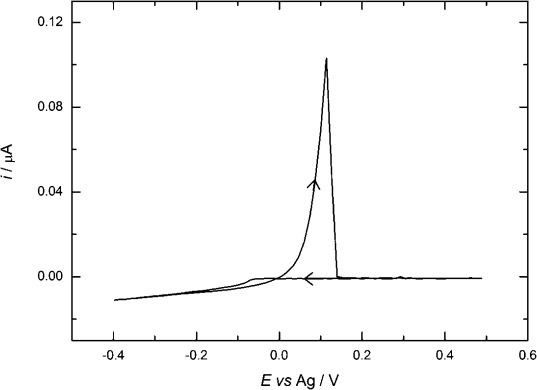
Cyclic voltammetry in 5 mm [Ag(hfac)(PPh_3_)] + 20 mm [*n*Bu_4_N][BF_4_] in acetonitrile solution for a 25 μm Pt working electrode. Recorded at a scan rate of 0.02 V s^−1^ and at a temperature of 297±1 K.

A typical cyclic voltammogram recorded at a 12.5 μm-radius Pt microdisk for[Ag(hfac)(PPh_3_)] in acetonitrile containing [*n*Bu_4_N][BF_4_] is shown in Figure 4. In this case, instead of the expected steady-state plateau, the current rises slowly with potential at higher cathodic potentials (beyond −0.1 V). This is not because of an increase in electrode area with deposition of the silver because the current decreases again on the return scan, suggesting that it is caused by some additional reduction process. This is confirmed by measurements of the Faradaic efficiency, which is only 0.75. We attribute this to the reduction of the triphenylphosphine ligand[28] (see the Supporting Information).

The interfering reduction reaction makes it difficult to measure the diffusion coefficient for the silver complex. Based on the value of the current at the start of the reduction wave, and by averaging the results from many experiments, we obtained an estimate of the diffusion coefficient at 297±1 K of (1.7±0.2)×10^−5^ cm^2^ s^−1^.

#### 2.2.4. Cyclic Voltammetry of [Ag(CF_3_(CF_2_)_6_CO_2_)(PPh_3_)_2_] in Acetonitrile

**Figure 5:**
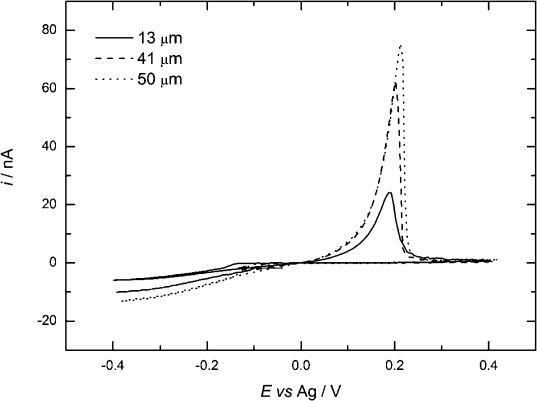
Cyclic voltammetry in 1 mm [Ag(CF_3_(CF_2_)_6_CO_2_)(PPh_3_)_2_] + 10 mm [*n*Bu_4_N][B{C_6_H_3_(CF_3_)_2_}_4_] in acetonitrile. Recorded at a scan rate of 0.05 V s^−1^ at Pt microelectrodes, and at a temperature of 297±1 K.

The silver(I) perfluorooctanoate di-triphenylphosphine complex was designed to solubilise the precursor in the low polarity SCF. Cyclic voltammetry in acetonitrile at microelectrodes of different sizes for 1 mm [Ag(CF_3_(CF_2_)_6_CO_2_)(PPh_3_)_2_] in 10 mm [*n*Bu_4_N][B{C_6_H_3_(CF_3_)_2_}_4_] is shown in Figure 5. Again, the current does not attain a plateau value, but it continues to increase slowly at more cathodic potentials; we attribute this to reduction of the triphenylphosphine ligand. The Faradaic efficiency of silver deposition was between 0.76 and 0.88, and the nucleation overpotential values were between 168 and 213 mV (see Table S2 in the Supporting Information). The diffusion coefficient for the silver complex was estimated to be 8.0×10^−6^ cm^2^ s^−1^ at 297±1 K.

#### 2.2.5. Cyclic Voltammetry of [Ag(PPh_3_)_4_][BF_4_] in Acetonitrile

**Figure 6:**
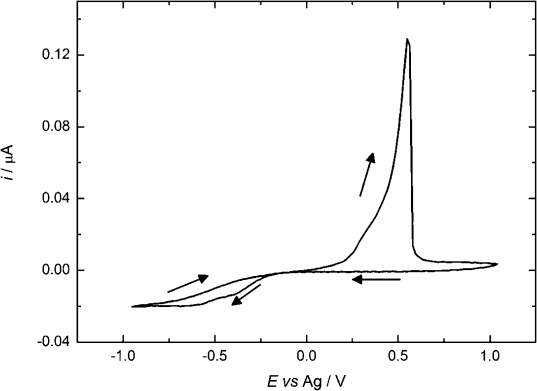
Cyclic voltammetry in 4.4 mm [Ag(PPh_3_)_4_][BF_4_] + 20 mm [*n*Bu_4_N][BF_4_] in acetonitrile at a 25 μm Pt disk. Recorded at a scan rate of 0.015 V s^−1^ and at a temperature of 297±1 K (first scan).

For completeness, we also investigated the electrochemistry of [Ag(PPh_3_)_4_][BF_4_]. Figure 6 shows the first scan recorded at a 12.5 μm Pt disk for 4.4 mm [Ag(PPh_3_)_4_][BF_4_] in acetonitrile containing 20 mm [*n*Bu_4_N][BF_4_] at room temperature. In this case, the voltammetry changes on repeated cycling, suggesting that the surface of the electrode is altered. On the first scan, there is no clear nucleation loop; however, on the second scan, a narrow nucleation loop is observed and on the third scan the difference in current values on the forward and backward scans in the loop is larger and the nucleation overpotential increases (see the Supporting Information, Figure S10). Based on the first scan data, we obtained an estimate of the diffusion coefficient of the silver complex of 9.4×10^−6^ cm^2^ s^−1^ at 297±1 K.

### 2.3. Electrochemistry in scCO_2_/CH_3_CN

The voltammetry of four complexes was examined in scCO_2_/CH_3_CN to compare with the behaviour in acetonitrile and to determine which complex or complexes are best-suited for silver deposition under supercritical conditions. The cyclic voltammograms were mostly recorded prior to bulk electrodeposition onto larger, flat or templated substrates. It is difficult to repeat experiments under exactly the same conditions, so there are slight variations in the pressure and temperature, as given in the Figure legends in each case.

#### 2.3.1. Cyclic Voltammetry of [Ag(CH_3_CN)_4_][BF_4_] in scCO_2_/CH_3_CN

**Figure 7:**
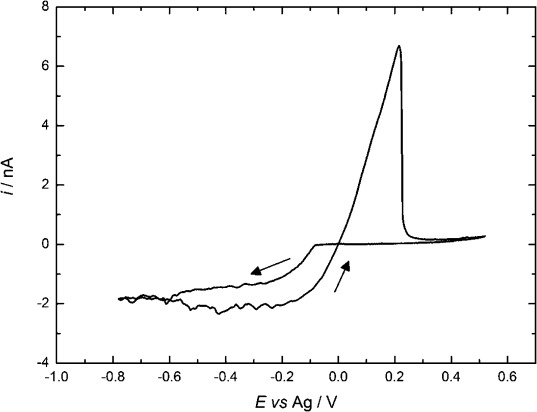
Cyclic voltammetry in 0.09 mm [Ag(CH_3_CN)_4_][BF_4_] in 20 mm [*n*Bu_4_N][BF_4_] scCO_2_/CH_3_CN (ca. 15 % v/v) at a 51 μm Pt disk at 310 K, 172 bar, and recorded at a scan rate of 0.02 V s^−1^.

Figure 7 shows an example of the voltammetry for [Ag(CH_3_CN)_4_][BF_4_] recorded in scCO_2_/CH_3_CN (for further examples see the Supporting Information). In all cases, features that are typical for cathodic metal deposition and anodic stripping are observed. The limiting deposition current at cathodic potentials often fluctuates in these experiments, and we attribute this to the effects of convection in the cell caused by temperature gradients, which are exacerbated by the low viscosity of the supercritical phase.

The potential difference between the reduction wave and stripping peak (Δ*E*_(eq−pa)_, see the Supporting Information Table S4) is increased from approximately 0.2 V in acetonitrile to 0.3 V in scCO_2_/CH_3_CN. This is accounted for by the fact that the silver stripping reaction requires acetonitrile2, and in scCO_2_/CH_3_CN, the concentration of acetonitrile is lower:


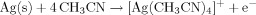
(2)

Diffusion coefficients, estimated from voltammograms on the first cathodic scan ranged from 6×10^−5^ to 36×10^−5^ cm^2^ s^−1^ and were larger than the estimated values in acetonitrile at room temperature (2.5×10^−5^ cm^2^ s^−1^) or at 308 K (3.0×10^−5^ cm^2^ s^−1^). The nucleation overpotential values (see Table S4 in the Supporting Information) range from 60 to 210 mV and increase with pressure. At the relatively low pressure of 90 bar and elevated temperature of 313 K, the overpotential was lowest, but the diffusion coefficient was also low under these conditions. The Faradaic efficiency for the silver plating, as judged by comparison of the deposition and stripping charges, was typically only 50 %, but we attribute this to poor adhesion of the deposit (solid particles were found in the reactor after the experiment) and significant lateral growth of silver outside the perimeter of electrode.

#### 2.3.2. Cyclic Voltammetry of [Ag(hfac)(COD)] in scCO_2_/CH_3_CN

**Figure 8:**
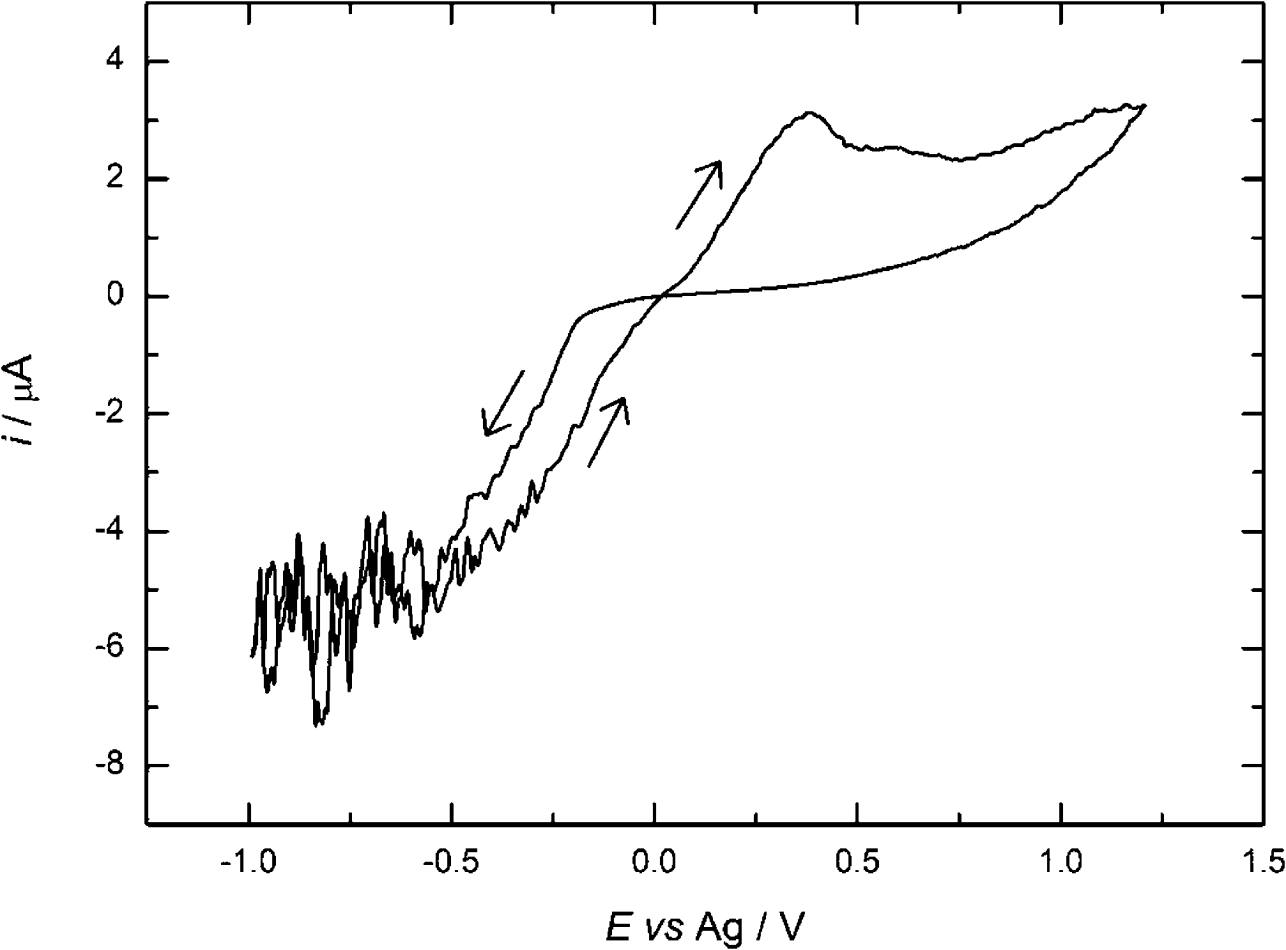
Cyclic voltammetry in 5.03 mm [Ag(hfac)(COD)] + 20.2 mm [*n*Bu_4_N][BF_4_] in scCO_2_/CH_3_CN (16.5 % v/v) at a 0.50 mm Pt disk, at 313 K, and at 144 bar.

Figure 8 shows the voltammetry for [Ag(hfac)(COD)] recorded at a 0.5 mm Pt disk in scCO_2_/CH_3_CN. The voltammogram shows evidence of some distortion caused by Ohmic loss (*iR* drop), owing to solution resistance. However, despite this, the nucleation loop and stripping peak can be seen. Again, the current at cathodic potentials is erratic, presumably caused by convection. The currents are relatively high and similar to those for [Ag(CH_3_CN)_4_][BF_4_].

When the reactor was disassembled after the experiments in the SCF, we observed excessive fouling of the counter electrode by a dark viscous oil. We attributed this to an electrochemically induced polymerisation reaction of the COD ligand. It is known that metal complexes with the COD ligand can act as catalysts in the cyclo-oligopolymerisation of alkynes and acetonitrile,[29] and it is possible that COD could be polymerised itself. This fouling of the counter electrode limits the utility of [Ag(hfac)(COD)] as a reagent for silver deposition in supercritical CO_2_/CH_3_CN.

#### 2.3.3. Cyclic Voltammetry of [Ag(hfac)(PPh_3_)] in scCO_2_/CH_3_CN

**Figure 9:**
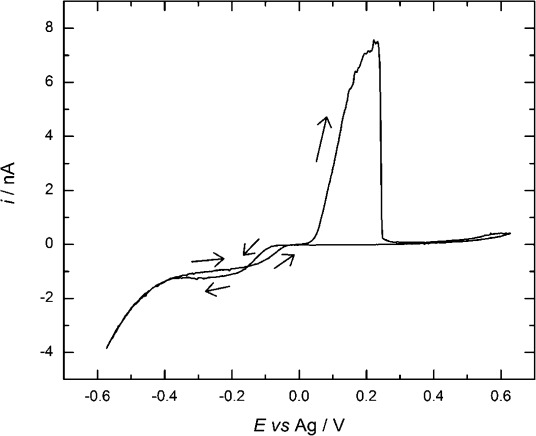
Cyclic voltammogram in 5.9 mm [Ag(hfac)(PPh_3_)] + 21.3 mm [*n*Bu_4_N][BF_4_] in scCO_2_/CH_3_CN (15.5 % v/v) at a 7.6 μm Pt/W (95:5) disk, at 316 K and 140 bar; recorded at a scan rate of 0.005 V s^−1^.

Figure 9 shows a cyclic voltammogram for [Ag(hfac)(PPh_3_)] in scCO_2_/CH_3_CN. Electrodeposition of silver is observed at −0.18 V versus Ag, with a further reduction wave starting beyond −0.4 V versus Ag. This second wave is not seen for the two previous complexes, and we conclude that it is caused by the reduction of triphenylphosphine coupled with a bulk catalytic reaction involving the tetrabutylammonium cation, as described above.[28] Again, the stripping peak in Figure 9 is noticeably broader than that for the corresponding process in acetonitrile solution (see Figure 4). The potential difference between the reduction wave and stripping peak increased from approximately 0.2 V in acetonitrile to 0.4 V in scCO_2_/CH_3_CN, because of the lower concentration of acetonitrile in the scCO_2_/CH_3_CN.

#### 2.3.4. Cyclic Voltammetry of [Ag(CF_3_(CF_2_)_6_CO_2_)(PPh_3_)_2_] in scCO_2_/CH_3_CN

**Figure 10:**
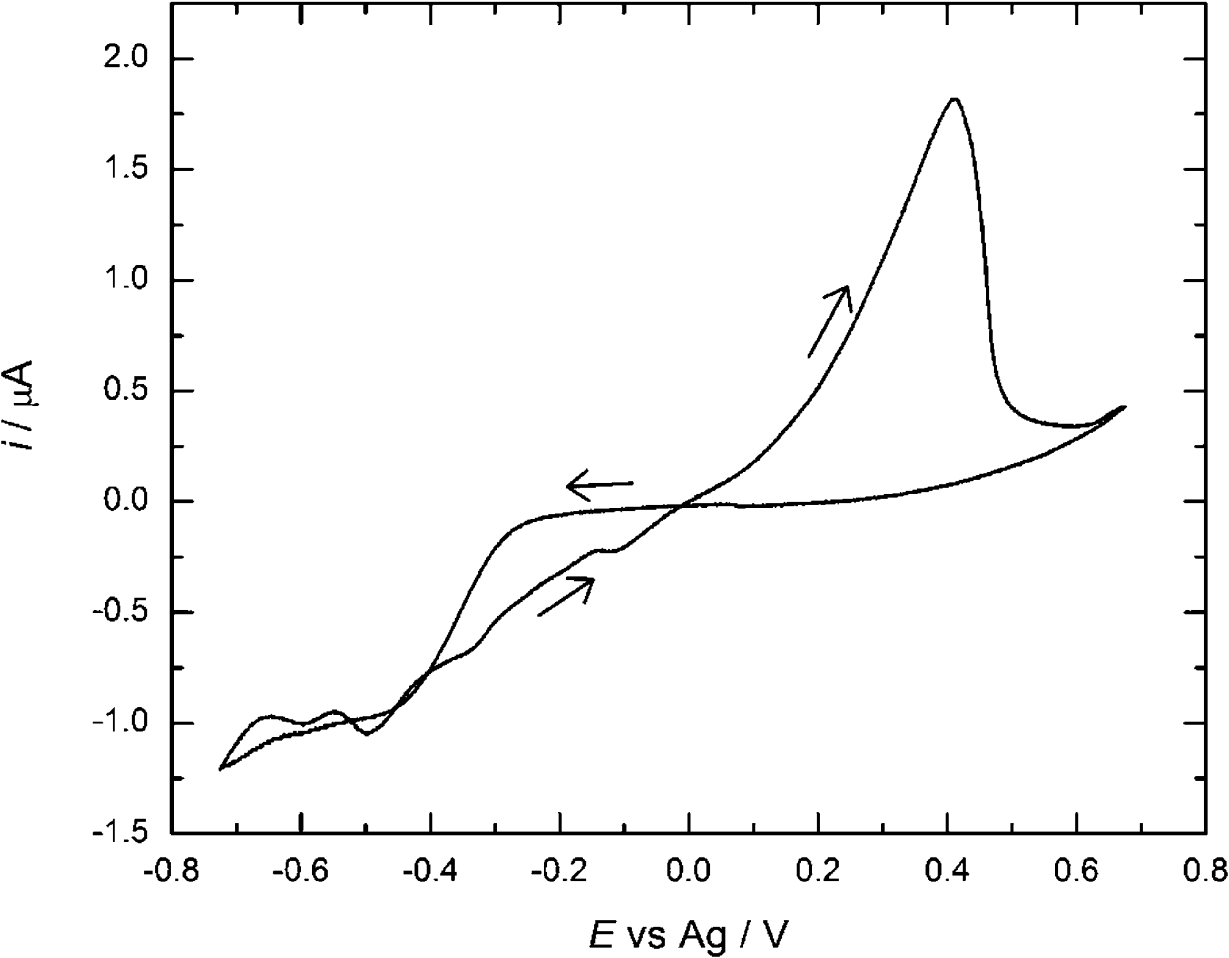
Cyclic voltammetry in 5 mm [Ag(CF_3_(CF_2_)_6_CO_2_)(PPh_3_)_2_] + 22 mm [*n*Bu_4_N][BF_4_] in scCO_2_/CH_3_CN (ca. 15 % v/v) at a 0.50 mm Pt disk electrode, at 312 K and 142 bar; recorded at a scan rate of 0.2 V s^−1^.

Figure 10 shows voltammetry for [Ag(CF_3_(CF_2_)_6_CO_2_)(PPh_3_)_2_] in scCO_2_/CH_3_CN. It shows the typical nucleation loop and stripping peak expected for silver deposition; however, at high cathodic potentials, the current continues to increase as a function of potential and we attribute this to the reduction of the triphenylphosphine ligand. The nucleation overpotential, 0.34 V, is significantly larger than the value in acetonitrile solution at room temperature (compare to Figure 5). The Faradaic efficiency for the deposition was 0.62.

### 2.4. Summary for Silver Precursors

All four silver complexes that were investigated were found to successfully deposit silver in supercritical CO_2_/CH_3_CN; however, they are not all equally suited as reagents for the deposition of films and nanowires. Solutions of the complexes were found to be stable in acetonitrile (when stored in a closed vessel in the dark) and gave reproducible voltammetric results over several days. Data for all complexes are given in Table S5 in the Supporting Information. The [Ag(hfac)(COD)] complex gave good silver deposition from supercritical CO_2_/CH_3_CN, but caused fouling of the counter electrode in the undivided cell. The complexes containing the PPh_3_ ligand, [Ag(CF_3_(CF_2_)_6_CO_2_)(PPh_3_)_2_] and [Ag(PPh_3_)_4_][BF_4_], also gave silver electrodeposition but, in this case, the voltammetry was complicated by the electrochemical reduction of the PPh_3_ ligand and its subsequent reaction with the electrolyte. For these reasons, the [Ag(CH_3_CN)_4_][BF_4_] precursor was chosen as the most suitable for silver electrodeposition from the SCF. It has a high diffusion coefficient in acetonitrile at room temperature (2.5×10^−5^ cm^−2^ s^−1^) and in scCO_2_/CH_3_CN (6–36×10^−5^ cm^2^ s^−1^ in the range 90–198 bar and 305-314 K). In addition, the reaction is clean, in the sense that no additional ligands are introduced into the solution, and no unwanted by products are generated in the reduction. For these reasons, the [Ag(CH_3_CN)_4_][BF_4_] was used in the subsequent studies of silver deposition.

### 2.5. Electrodeposition of Silver from scCO_2_/CH_3_CN

Silver films were electrodeposited from scCO_2_/CH_3_CN onto Pt macroelectrodes by using three of the silver complexes, [Ag(hfac)(COD)], [Ag(hfac)(PPh_3_)] and [Ag(CH_3_CN)_4_][BF_4_]. Results for the [Ag(hfac)(COD)] and [Ag(hfac)(PPh_3_)] complexes can be found in the Supporting Information. Here, we concentrate on the results for [Ag(CH_3_CN)_4_][BF_4_].

**Figure 11:**
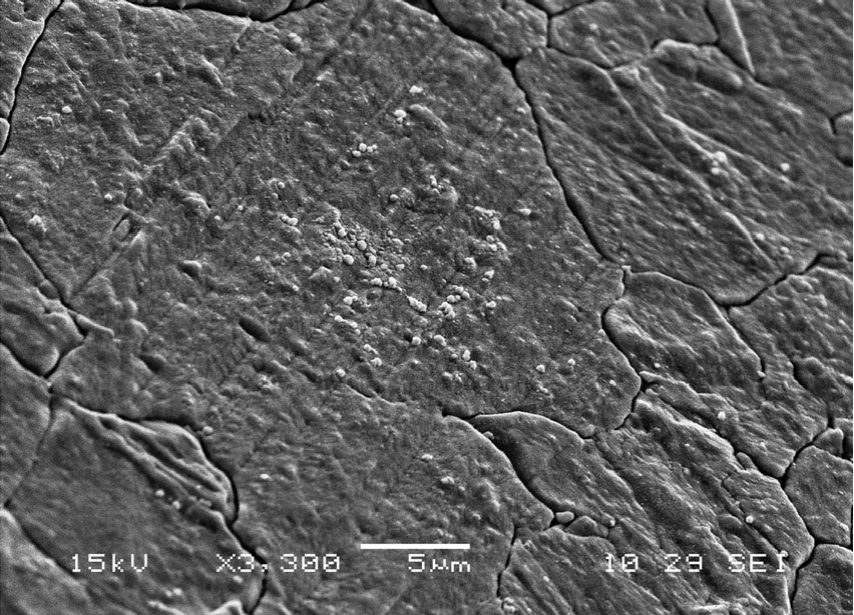
Field-emission gun (FEG)-SEM image of an approximately 10 μm-thick Ag film deposited on a Pt electrode from 6.3 mm [Ag(CH_3_CN)_4_][BF_4_] in 25 mm [*n*Bu_4_N][BF_4_] in scCO_2_/CH_3_CN (ca. 15 % v/v) at 318 K and 135 bar; deposition overpotential=0.05 V and deposition time=10 min.

Figure 11 shows SEM images of an approximately 10 μm-thick film of silver deposited from [Ag(CH_3_CN)_4_][BF_4_] in scCO_2_/CH_3_CN. The film had a silver-metallic appearance and was smooth with slight cracking. Energy-dispersive X-ray (EDX) analysis (not shown) indicates a silver film with a low impurity content.

#### 2.5.1. SCFED of Silver into AAO Membranes

Anodic alumina membranes provide a convenient hard template for the deposition of nanowires. For example, Zhang et al.[30] used magnetron sputtering into anodic alumina membranes to prepare 40–70 nm-diameter Ag nanowires. Kim et al.[31] used electroless deposition into anodic alumina to prepare silver nanowires with diameters between 20 and 200 nm; Sun et al.[32] and Sauer et al.[33] have reported electrodeposition of 100 nm- and 30–70 nm-diameter silver nanowires, respectively, into anodic alumina templates from aqueous solution.

**Figure 12:**
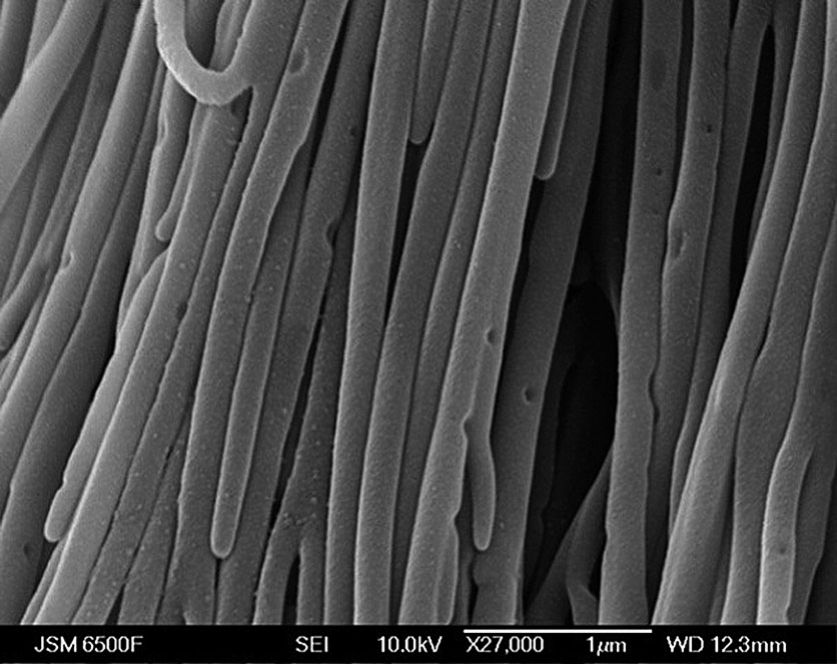
FEG-SEM image of silver nanowires on a partially dissolved 200 nm anodic alumina template (Whatman). The wires were deposited at an overpotential of 0.1 V from 4.8 mm [Ag(CH_3_CN)_4_][BF_4_] + 20 mm [*n*Bu_4_N][BF_4_] in scCO_2_/CH_3_CN at 309 K and 128 bar.

**Figure 13:**
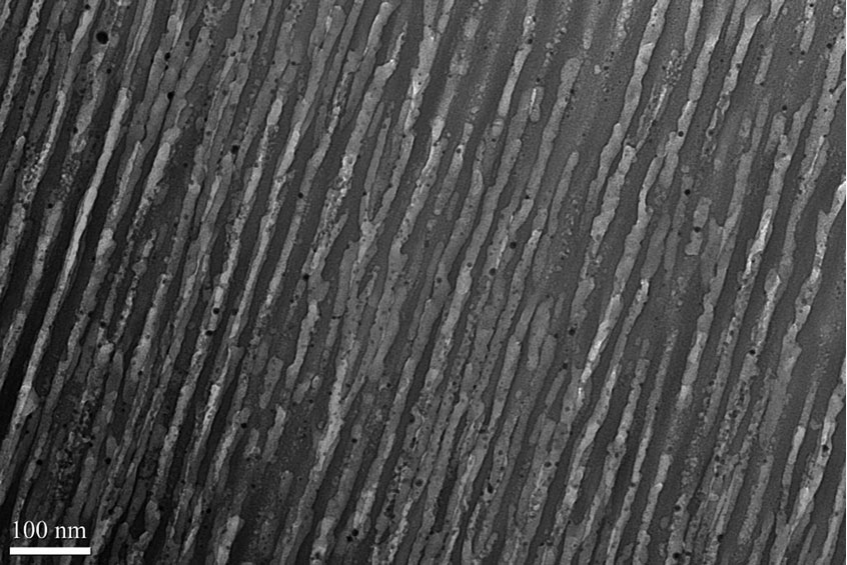
TEM image of silver nanowires deposited into an anodic alumina membrane (Synkera, thickness=60 μm, nominal pores size=13 nm) from 5 mm [Ag(CH_3_CN)_4_][BF_4_] + 20 mm [*n*Bu_4_N][BF_4_] in scCO_2_/CH_3_CN (12.1 wt %) at 310 K and 172 bar; deposited at −1 V versus Ag for 3600 s (charge, *Q*=0.872 C).

In the present work, three types of anodic alumina membranes with nominal pore diameters of 200, 20 and 13 nm and thicknesses of 60 μm were used. The images of the membranes before deposition are given in the Supporting Information. The 20 nm porous membrane showed a range of pore sizes from 20 to 200 nm, with an average pore diameter of approximately 40 nm. The pores of the 200 nm porous AAO were not uniform; it has been reported in the literature that the same Whatman membranes have pore diameters ranging from 150 to 350 nm.[34] Silver nanowires were successfully deposited from scCO_2_/CH_3_CN by using the [Ag(CH_3_CN)_4_][BF_4_] complex under a range of conditions. Figure 12 shows silver nanowires formed through electrodeposition in a nominally 200 nm porous alumina membrane. EDX analysis of the wires confirms that they are made of silver. Figure 13 shows a TEM image of 13 nm-diameter nanowires deposited into a Synkera anodic alumina membrane.

## 3. Conclusions

All five silver complexes investigated ([Ag(CH_3_CN)_4_][BF_4_], [Ag(hfac)(COD)], [Ag(hfac)(PPh_3_)], [Ag(CF_3_(CF_2_)_6_CO_2_)(PPh_3_)_2_] and [Ag(PPh_3_)_4_][BF_4_]) show characteristic voltammetry for silver deposition and stripping in acetonitrile. In the case of the complexes with triphenylphosphine ([Ag(hfac)(PPh_3_)], [Ag(CF_3_(CF_2_)_6_CO_2_)(PPh_3_)_2_] and [Ag(PPh_3_)_4_][BF_4_]) the silver reduction reaction is accompanied by reduction of the triphenylphosphine ligand, which complicates the voltammetry. For the four complexes studied in scCO_2_/CH_3_CN ([Ag(CH_3_CN)_4_][BF_4_], [Ag(hfac)(COD)], [Ag(hfac)(PPh_3_)] and [Ag(CF_3_(CF_2_)_6_CO_2_)(PPh_3_)_2_]), the voltammetry in the SCF is very similar to that in acetonitrile, with silver deposition and stripping occurring in all four cases.

The values of the diffusion coefficients found in the SCF are typically two to ten times greater than in acetonitrile at a comparable temperature. In the SCF, the reduction of the triphenylphosphine ligand again complicates the voltammetry for [Ag(hfac)(PPh_3_)] and [Ag(CF_3_(CF_2_)_6_CO_2_)(PPh_3_)_2_]. In the case of [Ag(hfac)(COD)] in scCO_2_/CH_3_CN, we find that the COD ligand reacts at the Pt counter electrode, forming a polymeric layer that restricts the electrochemistry. The best choice of the five reagents studied for silver electrodeposition from scCO_2_/CH_3_CN is, therefore, [Ag(CH_3_CN)_4_][BF_4_]. By using [Ag(CH_3_CN)_4_][BF_4_], we were able to deposit good quality silver films onto macroelectrodes from scCO_2_/CH_3_CN and to deposit silver nanowires with diameters down to 13 nm by deposition into anodic alumina templates.

## Supporting Information

As a service to our authors and readers, this journal provides supporting information supplied by the authors. Such materials are peer reviewed and may be re-organized for online delivery, but are not copy-edited or typeset. Technical support issues arising from supporting information (other than missing files) should be addressed to the authors.

miscellaneous_information
