# Supplementary material for: The Electrodeposition of Silver from Supercritical Carbon Dioxide/Acetonitrile
Source: ChemElectroChem. 2013 Nov 26;1(1):187–94. doi: 10.1002/celc.201300131 (PMC4964882; doi:10.1002/celc.201300131)
Supplement: Supplementary file 1 — miscellaneous_information [file celc0001-0187-sd1.pdf]

CHEMELECTROCHEM

Supporting Information

© Copyright Wiley-VCH Verlag GmbH & Co. KGaA, 69451 Weinheim, 2014

The Electrodeposition of Silver from Supercritical Carbon Dioxide/Acetonitrile

Philip N. Bartlett,*[a] Magdalena Perdjon-Abel,[a] David Cook,[a] Gillian Reid,[a] William Levason,[a] Fei Cheng,[a] Wenjian Zhang,[a] Michael W. George,[b] Jie Ke,[b] Richard Beanland,[c] and Jeremy Sloan[c]

celc_201300131_sm_miscellaneous_information.pdf

Supporting Information

1. Voltammetry in CH_3CN

1.1 Cyclic voltammetry of $[Ag(CH_3CN)_4][BF_4]$ (1) in CH_3CN

All cyclic voltammograms in acetonitrile solutions of $[Ag(CH_3CN)_4][BF_4]$ showed a steady state current, nucleation loop and stripping peak. Cyclic voltammograms at three different microelectrodes, in a 4.1 mM $[Ag(CH_3CN)_4][BF_4]$ solution in acetonitrile are presented in Fig. S1. The limiting current values increased with the size of the electrode. The correlation between the limiting current and electrode radius is shown in Fig. S2. It is linear as expected from the microelectrode equation.

The same series of measurements was made for 2.5 mM and 5.35 mM solutions of $[Ag(CH_3CN)_4][BF_4]$ in acetonitrile. The limiting currents, overpotentials, differences between the equilibrium and stripping peak potentials, and the average stripping to deposition charge ratios are given in Table S1. The diffusion coefficient estimated for the 2.5 mM solution was (2.62±0.31) x 10^{-5} cm^2 s^{-1}, and for the 5.35 mM solution it was (2.40±0.18) x 10^{-5} cm^2 s^{-1}, both at 297 K.

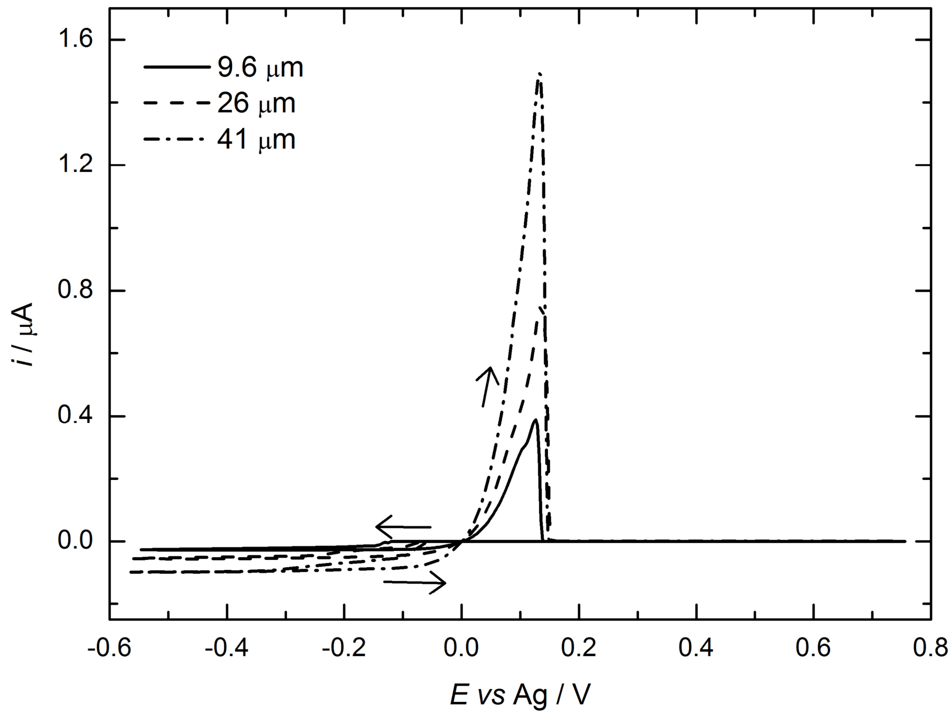

Fig. S1 Cyclic voltammograms in 4.1 mM $[Ag(CH_3CN)_4][BF_4]$ + 20 mM $[^nBu_4N][BF_4]$ in CH_3CN at microdisks of different sizes, recorded at 0.05 V s^{-1}, at room temperature.

Cyclic voltammograms for the same electrode in three solutions of different concentrations of $[Ag(CH_3CN)_4][BF_4]$ are shown in Fig. S2. The diffusion limited currents and stripping charges increases with the concentration as expected. The correlation between the limiting currents and concentrations is shown in Fig. S3. From the microdisk equation and the slope of the plot we obtain an estimate of the diffusion coefficient of (2.36±0.26) x 10^{-5} cm^2 s^{-1} at 297±1 K.

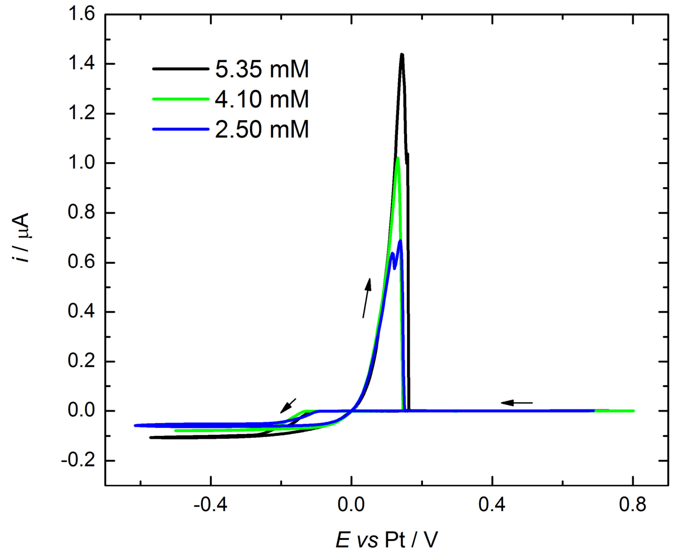

Fig. S2 Cyclic voltammograms in 2.5, 4.1 and 5.35 mM [Ag[(CH_3CN)$_4$][BF_4] + 20 mM [$^nBu_4N$][BF_4] in CH_3CN at a 20.6 μm radius Pt disk, recorded at 0.05 V s^{-1}, at room temperature.

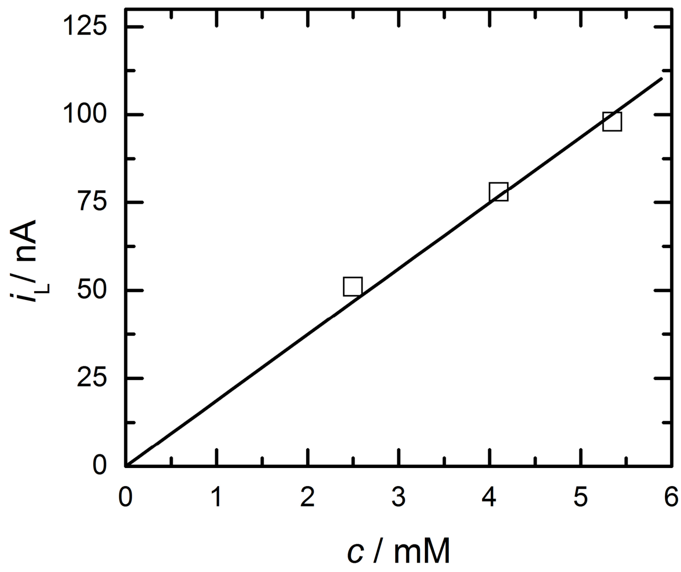

Fig. S3 Correlation between the limiting current and concentration of [Ag(CH_3CN)$_4$][BF_4] in 20 mM [$^nBu_4N$][BF_4] in CH_3CN at a 20.6 μm Pt disk, at room temperature. The estimated diffusion coefficient was (2.4 ± 0.3) x 10^{-5} cm^2 s^{-1}.

The experiments in the supercritical CO_2/CH_3CN are carried out at 308 K. Figure S4 shows a comparison of the voltammetry at 298 K and 308 K in CH_3CN.

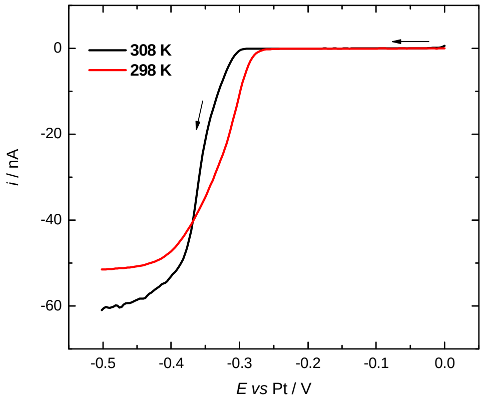

Fig. S4 The cathodic scan in 2.5 mM [Ag($CH_3CN)_4$][BF_4] + 20 mM [$^{n}Bu_4N$][BF_4] in CH_3CN at 298 and 308 K, at a 20.6 µm Pt disk.

The results for cyclic voltammetry of [Ag($CH_3CN)_4$][BF_4] in CH_3CN are summarised in Table S1.

Table S1 Data from cyclic voltammetry in [Ag($CH_3CN)_4$][BF_4] + 20 mM [$^{n}Bu_4N$][BF_4] in CH_3CN at microelectrodes, at 298 K and 308 K, at 0.05 V s^{-1}.

c / mM	a / µm	i_L / nA	η_{nuc} / mV	$\Delta E_{(eq\text{-}pa)}$ / mV	Average Q_{ox} / Q_{red}
5.35	4.8	26	134	145	0.93
	13.0	60	57	118	
	20.6	98	166	145	
	24.0	128	155	116	
4.10	4.8	20	139	125	0.97
	13.0	47	140	138	
	20.6	78	174	134	
	24.0	97	102	132	
2.50	4.8	12	170	99	0.94
	13.0	31	138	107	
	20.6	51	139	140	
	24.0	65	166	108	
2.50 (at 308 K)	20.6	60	87	86	0.94

1.2 Cyclic voltammetry of [Ag(hfac)(PPh_3)] (3) in CH_3CN

When the cathodic limit for [Ag(hfac)(PPh_3)] was extended, Fig. S5, an additional loop appears in the voltammogram (compare to Fig. 4 in text). We attribute the rising cathodic current beyond ca. -0.1 V vs Ag and the additional loop in the voltammetry to complications caused by the reduction of the triphenylphosphine ligand.

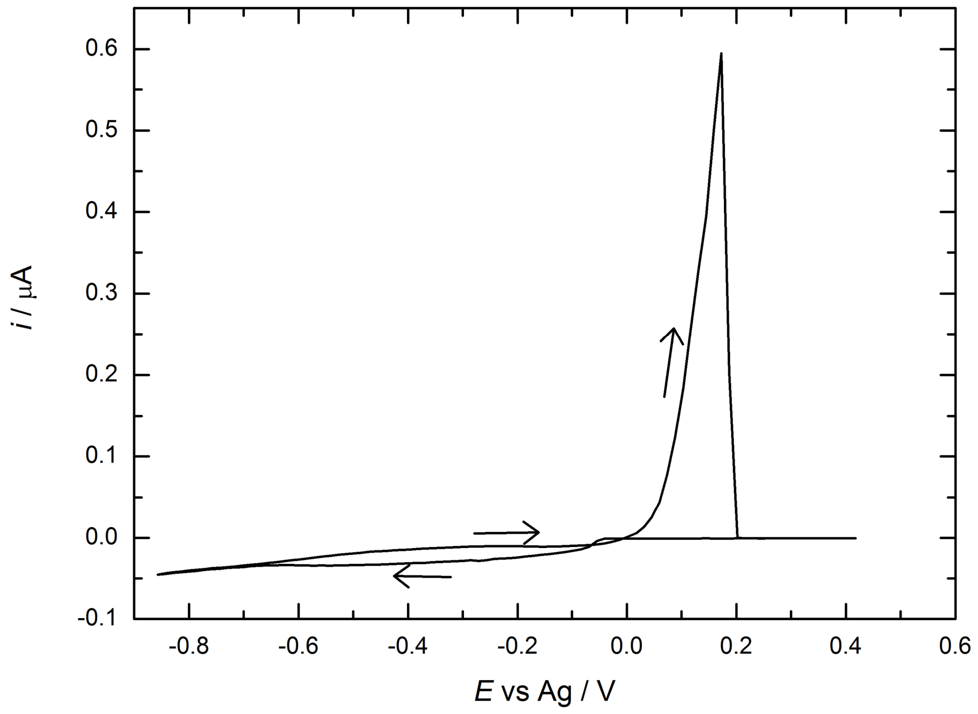

Fig. S5 Cyclic voltammetry in 5 mM [Ag(hfac)(PPh_3)] + 20 mM [^{n}Bu$_4$N][BF_4] in CH_3CN solution for a 25 μm Pt working electrode, recorded at 0.05 V s^{-1}, 297±1 K.

In order to verify the possible influence of the free PPh_3 in the solution, cyclic voltammetry was carried out on a 5 mM PPh_3 solution at a 12.5 μm Pt disk, Fig. S6. A large reduction wave was observed on the cathodic scan, followed by a nucleation loop, and a small stripping peak and an oxidation wave on the anodic scan. The charge of stripping (3.5 nC) corresponded to the oxidation of the species deposited at the electrode surface. This charge indicated the formation of a very thin (< 1 nm) layer of deposit.

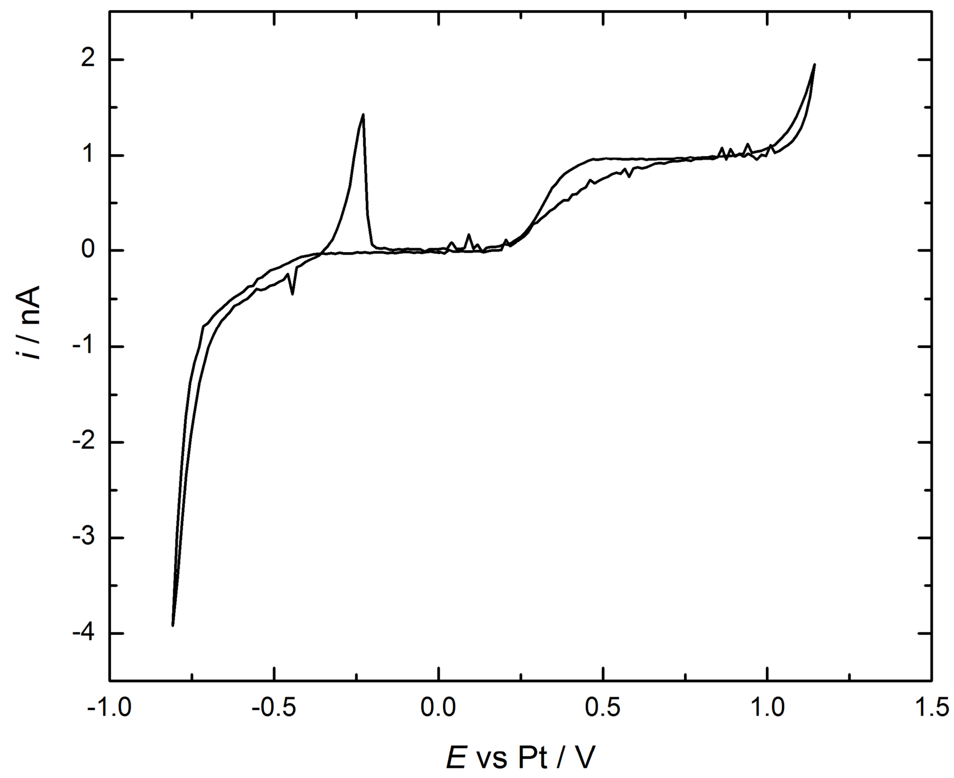

Fig. S6 Cyclic voltammogram of 5 mM PPh_3 + 20 mM [$^nBu_4N$][BF_4] in CH_3CN, recorded at 0.05 V s^{-1}, at a 25 µm Pt electrode, at 297±1 K.

The large reduction wave, which starts below -0.4 V vs. Pt corresponds to the reduction of triphenylphosphine.[1] Saveant and Binh reported that the electrochemical reduction of triphenylphosphine in acetonitrile leads to the catalytic reduction of the tetraalkylammonium cation of the supporting electrolyte.

$P\Phi_3 + e \rightarrow P\Phi_3^{-}$ (1)
$P\Phi_3^{-} + NBu_4^{+} \rightarrow BuP^{\bullet}\Phi_3 + NBu_3$ (2)
$BuP^{\bullet}\Phi_3 \rightarrow \Phi^{\bullet} + BuP\Phi_2$ (3)
$BuP^{\bullet}\Phi_3 \rightarrow Bu^{\bullet} + P\Phi_3$ (4)

In the mechanism they proposed,[1] the triphenylphosphine ($P\Phi_3$) is first reduced to an anion radical ($P\Phi_3^{-}$, reaction (1)). This anion radical carries out nucleophilic attack on the tetrabutylammonium cation forming a butyl-triphenylphoshine radical ($BuP^{\bullet}\Phi_3$) and tributylamine (NBu_3, reaction (2)). The radical continues to react in solution to produce, in one reaction, butyl-diphenylphosphine ($BuP\Phi_2$) and a phenyl radical ($\Phi^{\bullet}$, reaction (3)); and in a second reaction triphenylphosphine ($P\Phi_3$) and a butyl radical ($Bu^{\bullet}$, reaction (4)). The phenyl and butyl radicals can go on to react with the triphenylphosphine anion radical formed at the electrode to produce triphenylphosphine, phenyl anion, and butyl anion. The butyl radical can also dimerize in solution. This mechanism explains the catalytic effect of the triphenylphosphine and, in our experiments, the increasing current observed after the silver reduction wave.

The oxidation above 0.2 V vs. Pt in Fig. S6 corresponds to the formation of the triphenylphosphinium cation radical, which reacts with Ph_3P to form a phosphonium cation.[2] This reaction is followed by the oxidation of acetonitrile above 0.9 V vs. Pt.[3]

The products of the triphenylphosphine reduction followed by the catalytic process postulated by Saveant and Binh[1] (EC' mechanism) appear to adhere to the electrode surface.

In addition, during some of the experiments in acetonitrile, fine bits of white powder were observed in the bulk solution and the bottom of the glass cell. This was attributed to the formation of triphenylphosphine oxide ($OPPh_3$) by electrochemical oxidation of triphenylphosphine and by reaction with residual oxygen.[2, 4]

During the deposition of silver from [Ag(hfac)(PPh_3)] there will also be hexafluoroacetylacetonate (hfac) present in solution. The electrochemistry of the protonated ligand (Hfac) was examined in acetonitrile, Fig. S7. In the literature, it is reported that the reduction of Hfac in acetonitrile occurs at -0.97 V vs. Ag/Ag^+ and the oxidation at 1.09 V vs. Ag/Ag^+ (ΔE = 2.06 V).[5] In our experiments the reduction of Hfac was observed at -0.6 V vs. Pt, while the oxidation at approximately 1.4 V vs. Pt (ΔE = 2.0 V). The reduction of Hfac promotes the reduction of acetonitrile as the generated protons can react with acetonitrile to form either amine or imine type compounds.[6] This reduces the potential window.

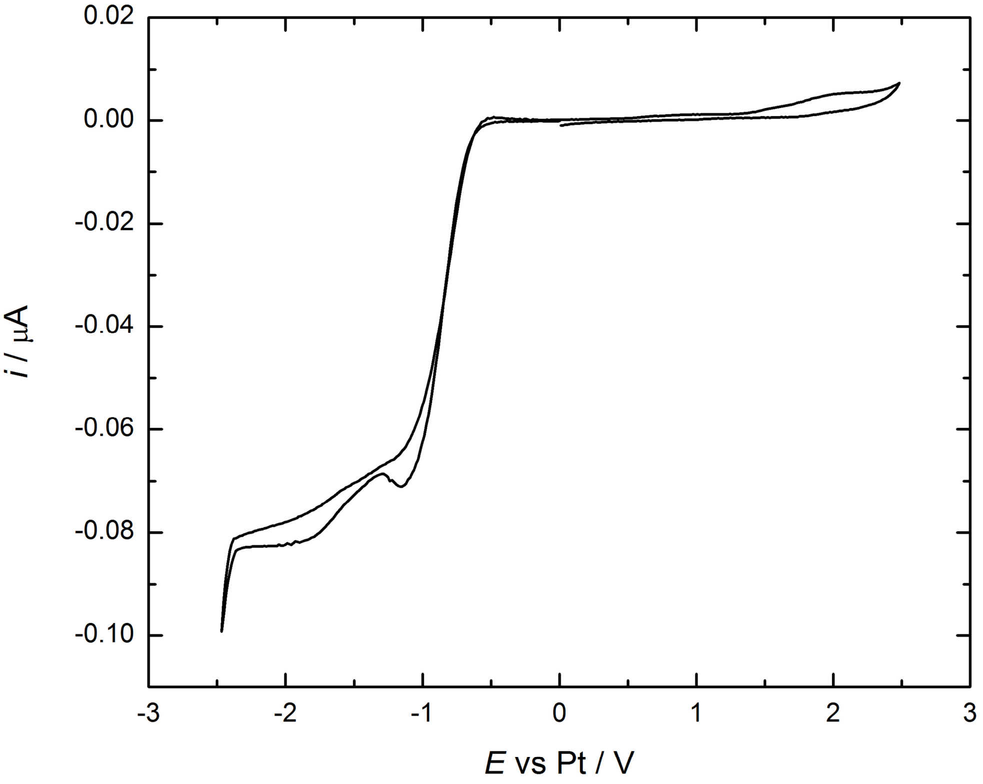

Fig. S7 Cyclic voltammogram of 5 mM Hfac + 20 mM [$^nBu_4N$][BF_4] in CH_3CN, at a 25 µm Pt electrode, recorded at 0.1 V s^{-1}, at 297±1 K.

1.3 Cyclic voltammetry of $[Ag(CF_3(CF_2)_6CO_2)(PPh_3)_2]$ (4) in CH_3CN

Figure S8 shows voltammetry for $[Ag(CF_3(CF_2)_6CO_2)(PPh_3)_2]$ in CH_3CN with 20 mM $[^nBu_4N][BF_4]$. These voltammograms are similar to those recorded with $[^nBu_4N][B\{C_6H_3(CF_3)_2\}_4]$ electrolyte shown in Figure 5 in the main text.

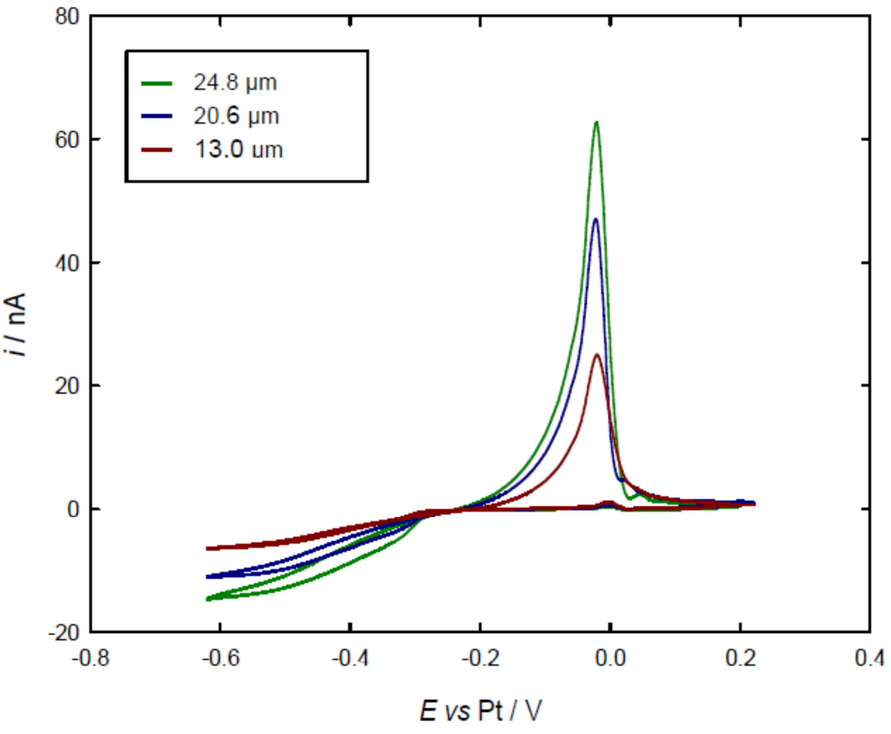

Fig. S8 Cyclic voltammetry in 1 mM $[Ag(CF_3(CF_2)_6CO_2)(PPh_3)_2]$ + 20 mM $[^nBu_4N][BF_4]$ in CH_3CN at 0.05 V s^{-1} for microdisk electrodes of 3 different sizes, at 297±1 K.

Cyclic voltammetry of $[Ag(CF_3(CF_2)_6CO_2)(PPh_3)_2]$ in acetonitrile with 0.1 M $[^nBu_4N][BF_4]$ is shown in Fig. S9. The main difference on increasing the supporting electrolyte concentration from 0.01 M to 0.1 M is the shift of the redox potential to more negative values. This is due to the changes in the potential of the Pt pseudoreference electrode.

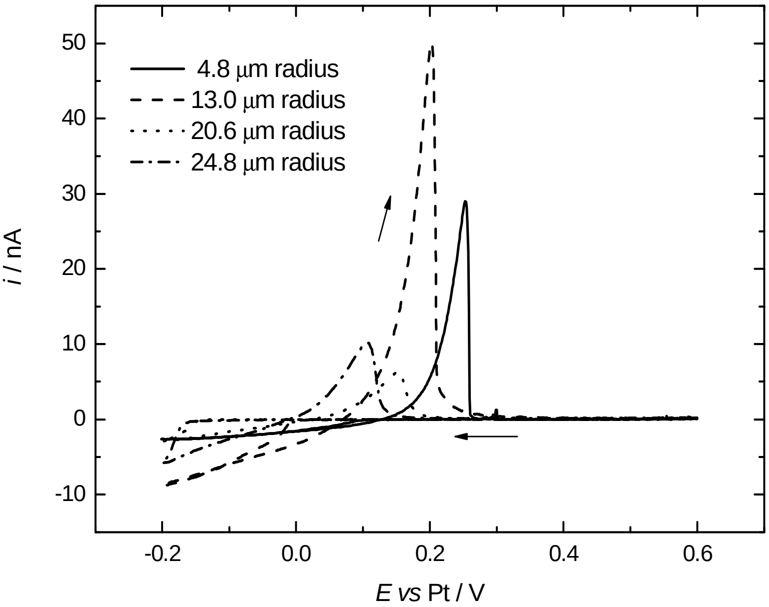

Fig. S9 Cyclic voltammetry in 1 mM [Ag(CF$_3$(CF$_2$)$_6$CO$_2$)(PPh$_3$)$_2$] + 0.1 M [^{n}Bu$_4$N][BF$_4$] in CH$_3$CN at 0.05 V s^{-1} at 4.8, 13.0, 20.6, and 24.8 μm Pt disk electrodes, at 297±1 K.

Table S2 Data from cyclic voltammetry in 1 mM [Ag(CF$_3$(CF$_2$)$_6$CO$_2$)(PPh$_3$)$_2$] + 10 mM [^{n}Bu$_4$N][B{C$_6$H$_3$(CF$_3$)$_2$}$_4$] in CH$_3$CN

***a* / μm**	***i*$_L$ / nA**	**η_{nuc} / mV**	**Q_{ox}/Q_{red}**
13.0	2.1	213	0.86
20.6	4.3	193	0.88
24.8	5.8	168	0.88

1.4 Cyclic voltammetry of [Ag(PPh$_3$)$_4$][BF$_4$] (5) in CH$_3$CN

The voltammetry of [Ag(PPh$_3$)$_4$][BF$_4$] in CH$_3$CN showed changes on repeated cycling. Figure S10 shows the third scan, compared to the first scan shown in Figure 6 of the main text the reduction wave is much more clearly defined and the overpotential increases significantly, Table S3.

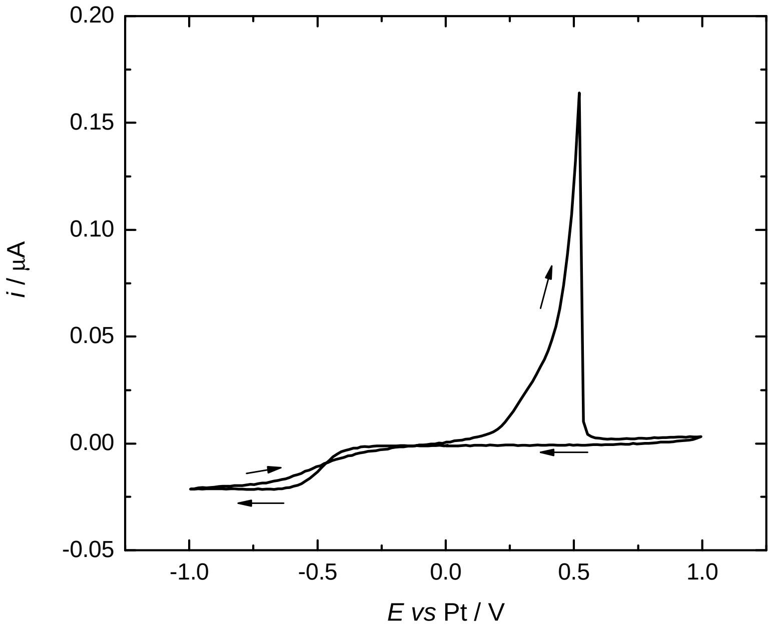

Fig. S10 Cyclic voltammetry in 4.4 mM $[Ag(PPh_3)_4][BF_4]$ + 20 mM $[^nBu_4N][BF_4]$ in CH_3CN at a 25 µm radius Pt disk, at 0.015 V s^{-1}, at 297±1 K, third scan.

Table S3 Data from cyclic voltammetry in 4.4 mM $[Ag(PPh_3)_4][BF_4]$ + 20 mM $[^nBu_4N][BF_4]$ in CH_3CN at room temperature

Scan	i_L / nA	η_{nuc} / mV	$\Delta E_{(eq\text{-}pa)}$ / V	Q_{ox}/Q_{red}
1	20.0	242*	0.639*	1.10
2	21.4	356	0.644	0.90
3	21.4	368	0.648	0.90

*the potential at which the forward and backward scan had the same current value, no nucleation loop was observed on the first scan

Note that the Faradaic efficiency for the first scan exceeded 100%, presumably due to some contribution from the triphenylphosphine (see above). The Faradaic efficiency remained stable at 90% for the second and consecutive cycles. The $\Delta E_{(eq\text{-}pa)}$ values were larger and the stripping peaks were broader than for other complexes.

2. Voltammetry in $scCO_2/CH_3CN$

2.1 Cyclic voltammetry of $[Ag(CH_3CN)_4][BF_4]$ (1) in $scCO_2/CH_3CN$

Further examples of cyclic voltammograms under different conditions in $scCO_2/CH_3CN$ at microelectrodes are presented in Figs. S11 to S16. The deposition current was often fluctuating, which suggests a weak adherence of the deposited silver to the substrate further resulting in low Faradaic efficiencies. Significant overgrowth was observed at slow scan rates, Figs. S11 and S15, as demonstrated by the rising plateau current. Data obtained from cyclic voltammetry in $[Ag(CH_3CN)_4][BF_4]$ solutions in $scCO_2/CH_3CN$ are listed in Table S4. The steady-state current was

approximated to the current at the base of the reduction wave. These currents most probably contain migration contributions (low conducting medium) and in some cases contribution from convection (e.g. resulting from uneven temperature in the cell and the low solvent viscosity).

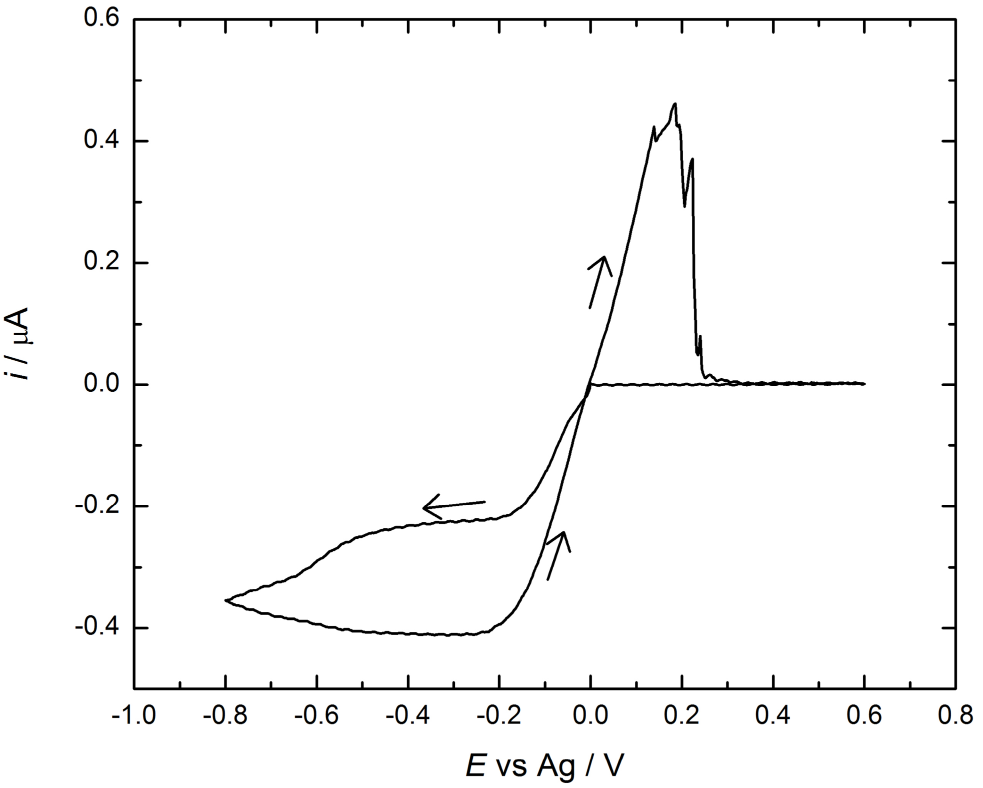

Fig. S11 Cyclic voltammetry in 4.65 mM [$Ag(CH_3CN)_4$][BF_4] + 19.6 mM [$^nBu_4N$][BF4] in $scCO_2/CH_3CN$ (15%$_{v/v}$) at 308 K and 138 bar, for a 7.6 μm Pt/W disk, recorded at 0.05 V s^{-1}.

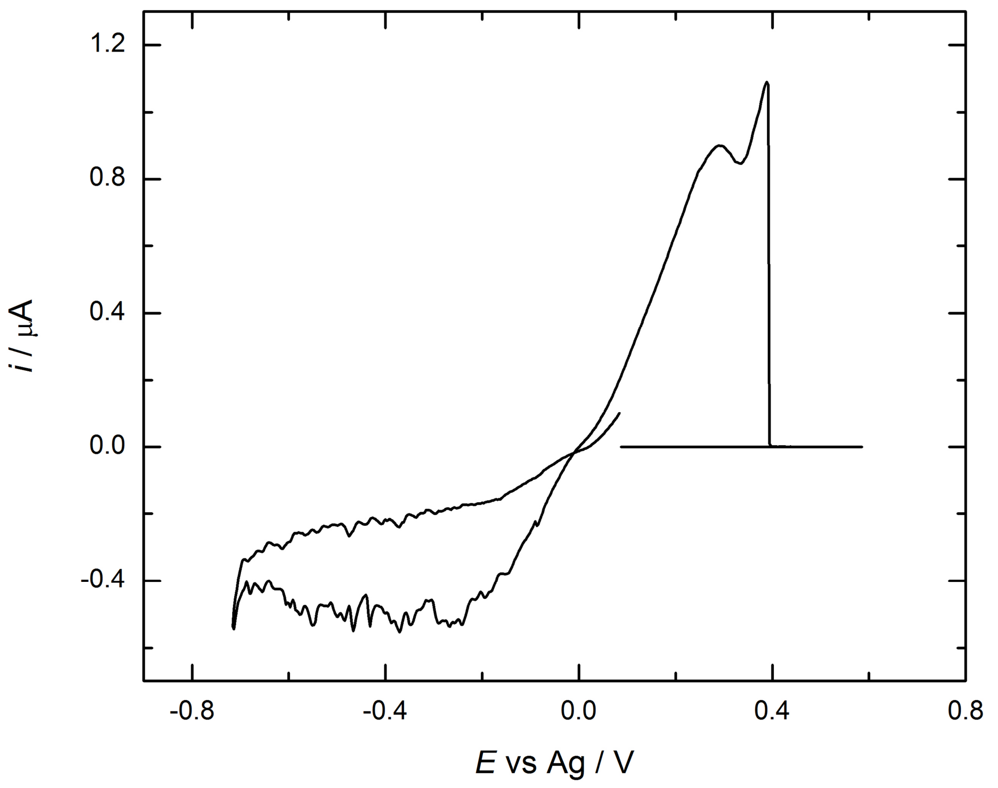

Fig. S12 Cyclic voltammetry in 5.3 mM [Ag(CH_3CN)$_4$][BF_4] + 20.3 mM [$^nBu_4N$][BF_4] in $scCO_2/CH_3CN$ (15.3%$_{v/v}$) at 307 K and 133 bar, for a 7.6 µm Pt/W disk, recorded at 0.02 V s^{-1}.

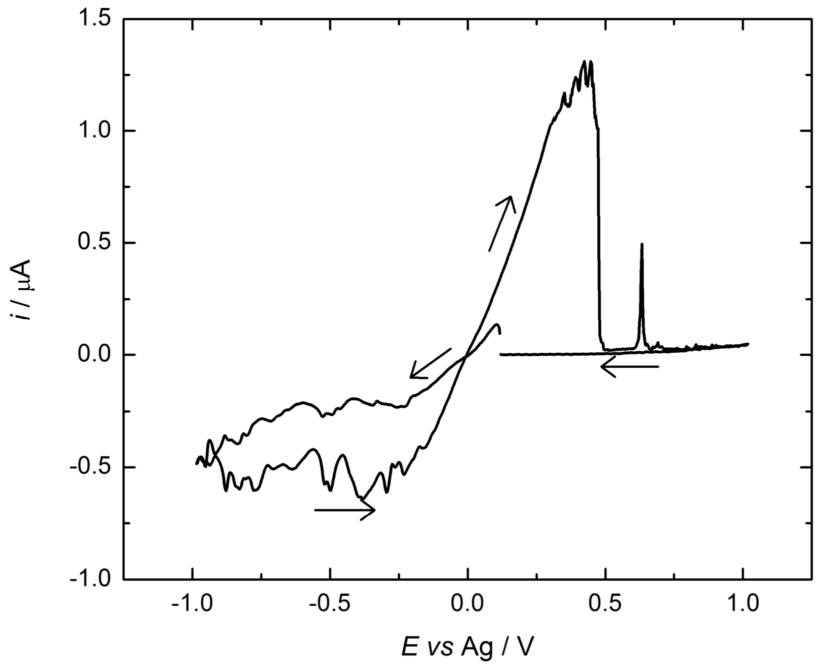

Fig.S 13 Cyclic voltammetry in ~5 mM [Ag(CH_3CN)$_4$][BF_4] + ~20 mM [$^nBu_4N$][BF_4] in $scCO_2$/CH_3CN (~15%$_{v/v}$) at 305 K and 194 bar, for a 31 µm Pt/W disk, recorded at 0.05 V s^{-1}.

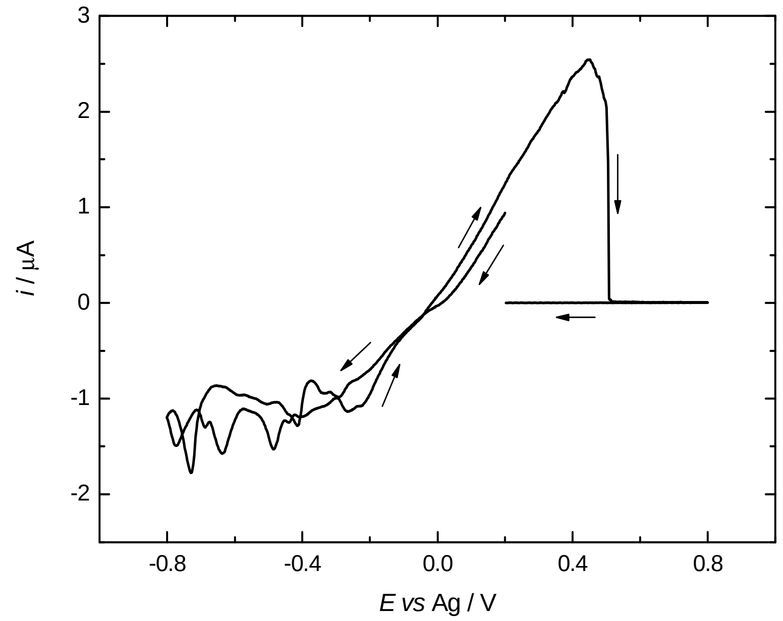

Fig. S14 Cyclic voltammetry in 7.1 mM [Ag(CH_3CN)$_4$][BF_4] + 19.5 mM [$^nBu_4N$][BF_4] in $scCO_2$/CH_3CN (15%$_{v/v}$), at 309 K and 166 bar, at a 25 µm Pt disk, recorded at 0.05 V s^{-1}.

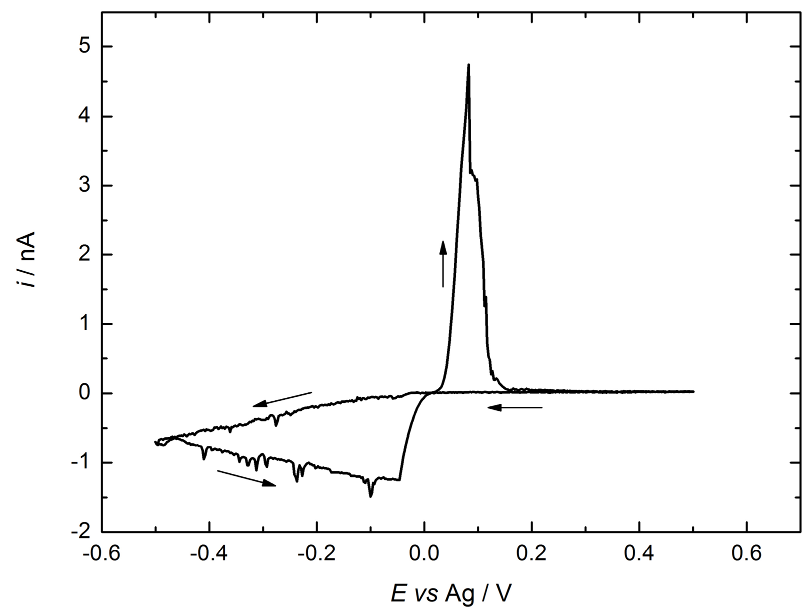

Fig. S15 Cyclic voltammetry in 3 mM $[Ag(CH_3CN)_4][BF_4]$ + 10 mM $[^nBu_4N][BF_4]$ in $scCO_2/CH_3CN$ ($15\%_{v/v}$) at 313 K and 164 bar, at a 7.6 µm Pt disk, recorded at 0.01 V s^{-1}.

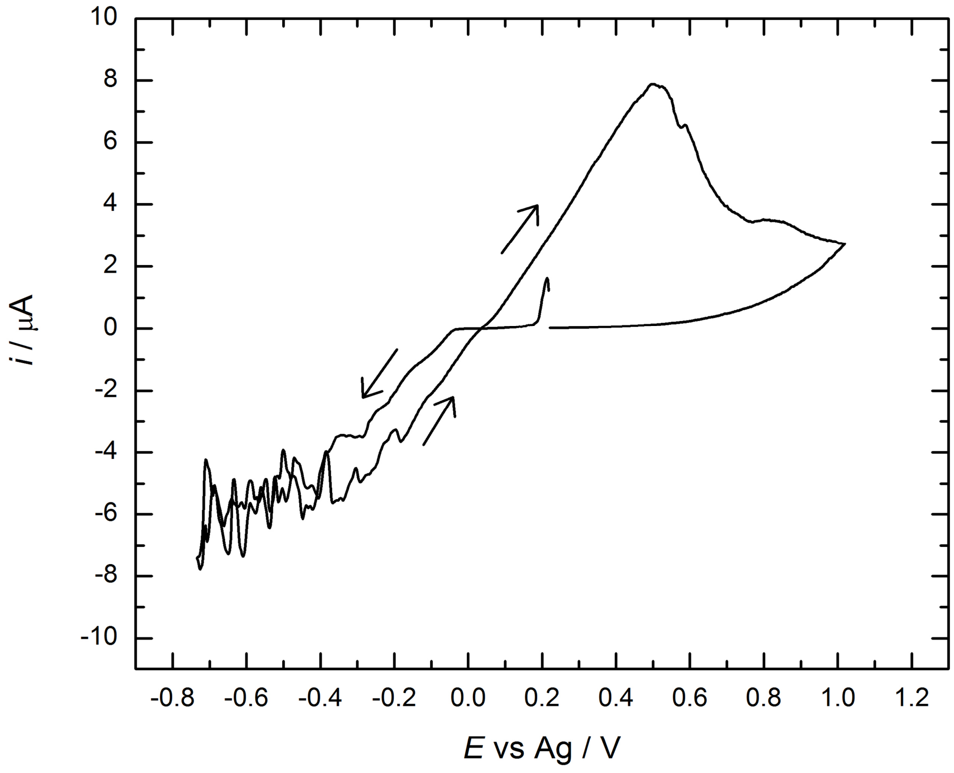

Fig. S16 Cyclic voltammetry in 6.56 mM [Ag(CH_3CN)$_4$][BF_4] + 26 mM [$^nBu_4N$][BF_4] in $scCO_2/CH_3CN$ (16.7 $\%_{v/v}$) at 318 K and 134 bar at a 0.50 mm Pt disk, at 0.02 V s^{-1}.

Apparent diffusion coefficients were estimated with the assumption that the current at the bottom of the reduction wave (not applicable in some cases, e.g. Fig. S15) was wholly limited diffusion and not under mixed diffusion/migration/convection control. Their values range from 6.1 x 10^{-5} to 35.5 x 10^{-5} cm^2 s^{-1}, and are all higher than the estimated (2.5±0.3) x 10^{-5} cm^2 s^{-1} for acetonitrile solution at room temperature and 3.0 x 10^{-5} cm^2 s^{-1} for acetonitrile solution at 308 K. There is not sufficient data to distinguish any significant trends relating to temperature, pressure or electrolyte concentration. Table S4 also gives the values of nucleation overpotential, and the differences between the equilibrium and anodic peak potentials, $\Delta E_{(eq\text{-}pa)}$. The nucleation overpotential values range from 61 to 208 mV, and increases with pressure. The system at a relatively low pressure of 90 bar and high temperature (in this work) of 313 K had the lowest electrodeposition overpotential but also one of the lowest diffusion coefficients. In the supercritical fluid, unlike the acetonitrile solutions, all overpotential values were lower than the respective $\Delta E_{(eq\text{-}pa)}$ values which were in the range 120 and 484 mV indicating that a more positive potential was required to strip the silver deposit off than in acetonitrile. This is not surprising because the stripping reaction required CH_3CN ligands for the Ag^+ and there are only approximately 15$\%_{v/v}$ of the supercritical. The Faradaic efficiency was only between 21 and 60%. We attribute this to two reasons: first the poor adhesion of deposit at the electrode surface (small particles of shiny material were found in the reactor at the end of the experiment), and second to significant growth of the deposit beyond the perimeter of electrode.

Table S4 Data from cyclic voltammetry of $[Ag(CH_3CN)_4][BF_4]$ (**1**) in $scCO_2/CH_3CN$

p / bar	*T* / K	c_{Ag} / mM	$c_{supp.el.}$ / c_{Ag} support ratio	$\%_{v/v}$ CH_3CN	*a* / µm	i_L / nA	*D* x 10^{-5} / $cm^2\ s^{-1}$	η_{nuc} / mV	$\Delta E_{(eq-pa)}$ / mV	Q_{ox}/ Q_{red}
90	313	0.14	143	15.0	15.6	5.6	6.84	61	120	0.21
124	314	5.20	3.81	16.0	15.6	337	10.8	78	320	0.24
117	308	4.65	4.21	15.0	3.8	223	32.7	77	196	0.14
133	307	5.30	3.83	15.3	3.8	230	29.6	105	405	0.51
148	308	7.50	2.63	14.5	3.8	390	35.5	75	244	0.22
166	309	5.10	2.75	14.9	4.8	107	11.3	98	484	0.55
172	310	0.09	222	15.0	3.8	1.44	33.0	141	215	0.36
179	312	1.00	20.0	15.0	4.8	11.3	6.1	208	278	0.60
194	305	5.00	4.0	15.0	15.6	226	7.5	119	434	0.55
198	307	0.10	200	15.0	25	27	28.0	150	-	0.22

2.2 Summary

Table S5 summarises the results for the voltammetry of the different complexes in CH_3CN and $scCO_2/CH_3CN$.

Table S5 Diffusion coefficients (room temperature and SCF), Faradaic efficiencies, and average potential differences for silver precursors in CH_3CN and $scCO_2/CH_3CN$

Precursor	Solvent	*D* x 10^{-5} / $cm^2\ s^{-1}$	Q_{ox}/Q_{red}	η_{nuc} / mV	$\Delta E_{(eq-pa)}$ / mV
$[Ag(CH_3CN)_4][BF_4]$ (**1**)	CH_3CN	2.5 ± 0.31	0.95	57 – 174	126
	$scCO_2$/ CH_3CN 90-198 bar 305 – 314 K	6.1 – 35.5	0.60	111	270
[Ag(hfac)COD] (**2**)	CH_3CN	2.2 ± 0.20	0.99	59	1128
$[Ag(hfac)(PPh_3)]$ (**3**)	CH_3CN	1.7 ± 0.20	0.75	93	121
	$scCO_2$/ CH_3CN 140 bar 315 K	-	0.72	119	246
$[Ag(CF_3(CF_2)_6CO_2)(PPh_3)_2]$ (**4**)	CH_3CN	0.8 ± 0.12	0.83	197	210
	$scCO_2$/ CH_3CN 142 bar 312 K	-	0.61	339	410
$[Ag(PPh_3)_4][BF_4]$ (**5**)	CH_3CN	0.94 ± 0.15	0.90	242	639

3. Ag deposition from $scCO_2/CH_3CN$

3.1 Electrodeposition of Ag from [Ag(hfac)(COD)] (2)

An example of a transient for electrodeposition from [Ag(hfac)(COD)] in $scCO_2/CH_3CN$ is shown in Fig. S17. The transient currents were erratic and fluctuated around a maximum, which indicated a limitation to the deposition process. The current was limited probably due to the products of the counter reaction blocking the platinum counter electrode. The product of the counter reaction was a dark deposit consisting of C, F and O. If the deposit insulated the parts of the counter electrode that it covered, it would limit the rate of silver deposition. The erratic transients could also be a result of the formation an insulating layer and/or convection in the supercritical fluid.

The electrodeposition times from the [Ag(hfac)COD] precursor solutions in the scf were kept short (2-3 min) due to the excessive formation of the by-product on the counter electrode.[7]
The SEM images of the 2.7 micron thick deposit are presented in Figs. S17b-e. The deposit consists of a uniform layer with larger 0.5-2 µm diameter grains on top of it. The EDX analysis of the silver deposit on platinum is presented in Fig. S17f and indicates a film with low impurity content.

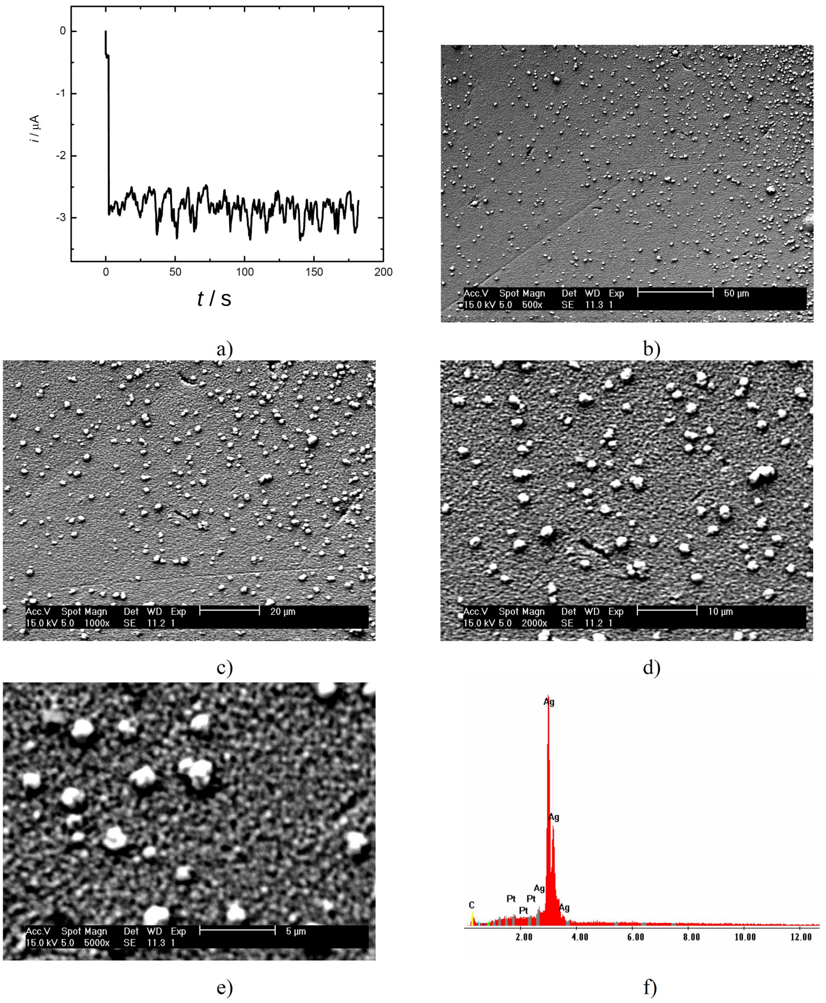

Fig. S17 Electrodeposition of Ag at a 0.5 mm Pt disk from 6 mM [Ag(hfac)(COD)] + 24 mM $[^nBu_4N][BF_4]$ in $scCO_2/CH_3CN$ ($15.8\%_{v/v}$), at an overpotential of 0.1 V, at 311 K and 109 bar, a) deposition transient, b-e) SEM images of the 2.7 µm thick film deposited in 3.6 min, f) EDX of deposit at x5000 magnification.

3.2 Electrodeposition of silver from [Ag(hfac)(PPh_3)] (3)

The electrodeposition transients, SEM images and EDX analysis for two silver deposits from the scf are presented in Figs. S18 and S19. The first deposition was carried out at 307 K and 127 bar, while the other at 317 K and 150 bar, at the same deposition overpotential of 0.1 V. The transient features for both are similar with the current continuously increasing from the start of deposition, with the deposition rate higher at the higher temperature. The EDX analyses of the deposits in Figs. S18b and S19b, show no impurities at the level of detection of the instrument. The SEM images of the two deposits, Figs. S18c,d and S19c-f, show smooth films with grains below a micron in size and particles up to 5 microns in diameter randomly distributed on top of the films.

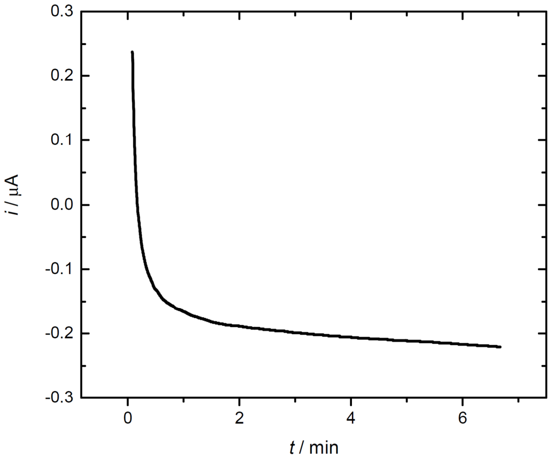

a)

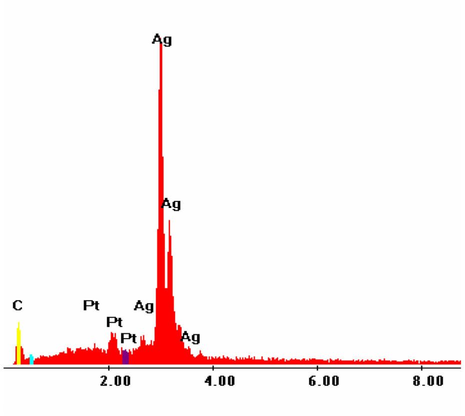

b)

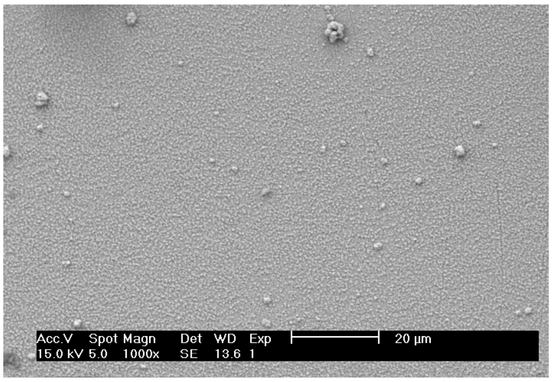

c)

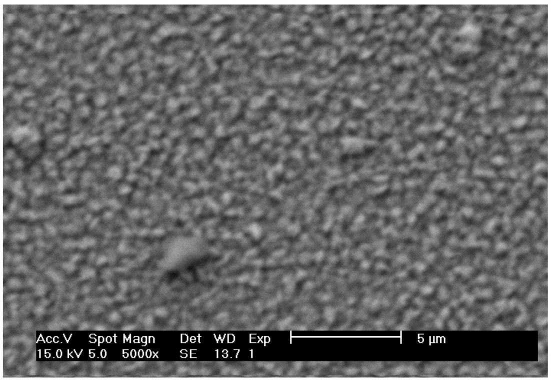

d)

Fig. S18 Electrodeposition of silver from 5 mM [Ag(hfac)(PPh_3)] + 20 mM [$^nBu_4N$][BF_4] in $scCO_2/CH_3CN$ (~15%$_{v/v}$), at a deposition overpotential of 0.1 V, at 307 K and 127 bar, on a 0.5 mm Pt disk, a) transient, b) EDX analysis of the deposit, c), d) SEM images at different magnifications.

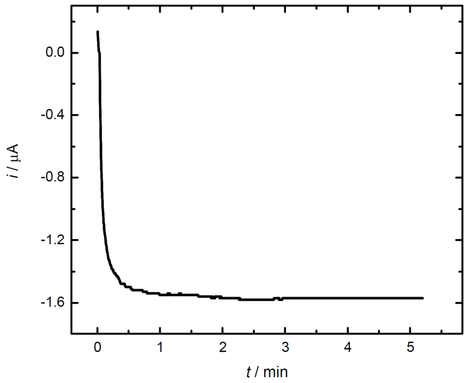

a)

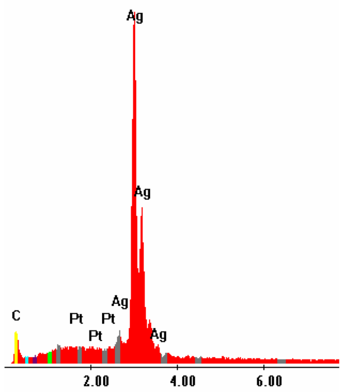

b)

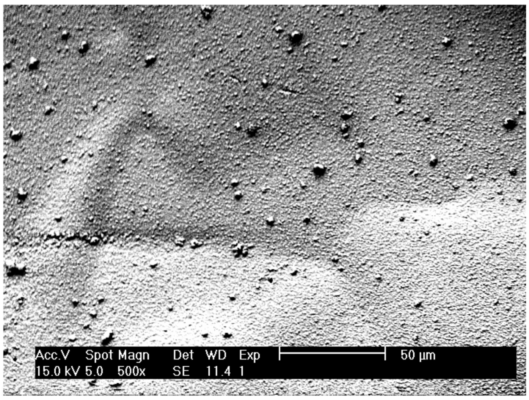

c)

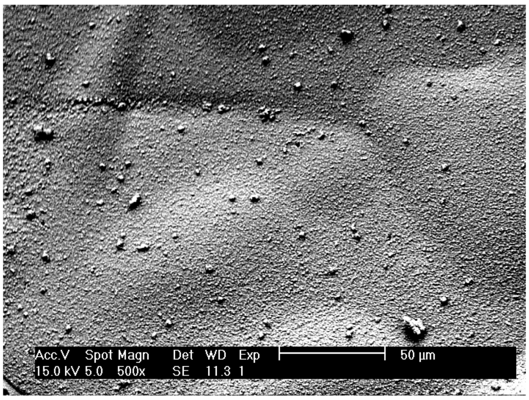

d)

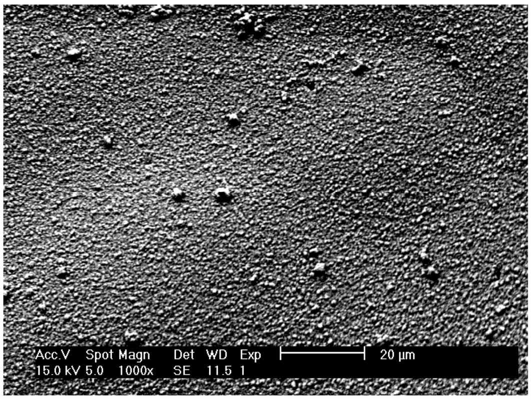

e)

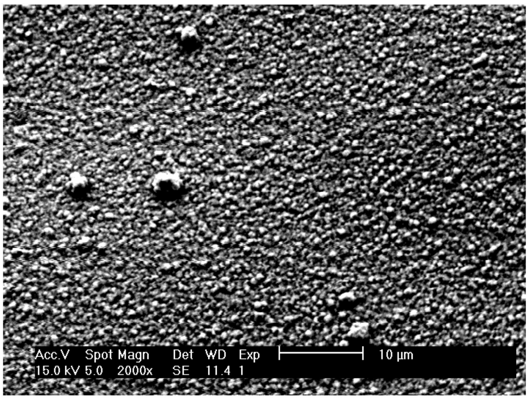

f)

Fig. S19 Electrodeposition of silver from 5 mM [Ag(hfac)(PPh_3)] + 20 mM [$^n Bu_4N$][BF_4] in $scCO_2/CH_3CN$ (~15%$_{v/v}$), at a deposition overpotential of 0.1 V, at 317 K and 150 bar, on a 0.5 mm Pt disk, a) transient, b)-e) SEM images at different magnifications, and f) EDX analysis of the deposit.

These particles are much fewer than those seen on the deposit from [Ag(hfac)(COD)].

3.3 Summary

The above and other data obtained from the SCFED experiments with the three silver complexes at 0.5 mm platinum and silver disks are presented in Table S6. The table contains details of deposition conditions, charges passed during deposition, estimated film heights and average growth rates. The concentration of silver complexes in the sc solutions was approximately 5-6 mM. The acetonitrile content was between 15 and 17 $\%_{v/v}$ (12 and 13.4 %w), the temperatures varied from 304 to 320 K, and pressures were between 105 and 152 bar.
Silver films were deposited at Pt and Ag working electrodes, Table S6. The film growth rate was higher in [Ag(hfac)COD] and $[Ag(CH_3CN)_4][BF_4]$ solutions comparing to the $[Ag(hfac)(PPh_3)]$. The PPh_3 ligand is electrochemically active in the silver deposition range significantly reducing the deposition efficiency and complicating the deposition process.

Table S6 Data from the electrodeposition of silver $scCO_2/CH_3CN$ at 0.5 mm Pt and Ag disk electrodes

Precursor	T / K	p / bar	WE	η / V	t_{dep} / min	Q / mC	h^c / µm	v^d / µm min^{-1}
[Ag(hfac)COD] (**2**)[a]	314	145	Pt	0.30	2	0.4	2.17	1.08
				0.20	2	0.3	1.63	0.81
	307	142		0.10	3	0.5	2.72	0.91
	311	127		0.10	3	0.5	2.72	0.91
$[Ag(hfac)(PPh_3)]$ (**3**)	304[b]	125	Pt	0.05	10	1.4	7.61	0.76
	306	124		0.05	10	1.1	5.98	0.60
	316	150		0.10	5	0.49	2.66	0.53
				0	5	0.30	1.63	0.33
	316	120		0	10	0.35	1.90	0.19
			Ag	0	10	0.42	2.28	0.23
$[Ag(CH_3CN)_4][BF_4]$ (**1**)	318	134	Pt	0	10	1.85	10.1	1.0

[a] deposition limited by counter electrode reaction; [b] near critical state; [c] deposition film thickness; [d] average deposition rate

3.4 SCFED of silver into AAO membranes

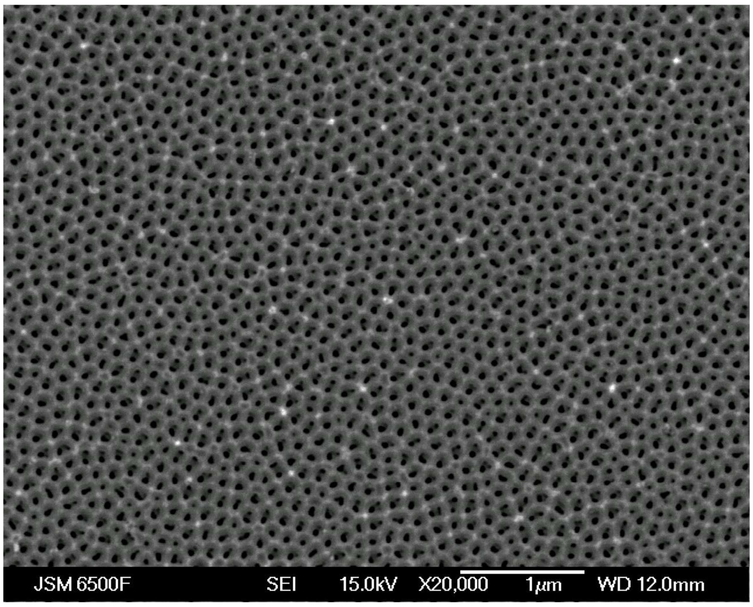

Fig S20 SEM of anodic alumina membranes (Synkera) with nominally 50 nm pores (as assessed by Synkera using gas permeance measurements). Samples were sputtered with 2-5 nm of gold before imaging in a JEOL JSM 6500F field emission gun microscope.

References

[1] J. M. Saveant, S. K. Binh, *J. Electroanal. Chem.* **1978**, *88*, 27.

[2] G. Schiavon, S. Zecchin, G. Cogoni, G. Bontempelli, *J. Electroanal. Chem.* 1973, *48*, 425.

[3] J. Zieja, J. Gadomska-Trzos, Z. Stojek, *Electroanal.* **2001**, *13*, 621.

[4] a) K. S. V. Santhanam, A. J. Bard, *J. Am. Chem. Soc.* **1967**, *90*, 1118; b) H. Ohmori, S. Nakai, M. Masui, *J. Chem. Soc. Perkin I* **1978**, *7*, 1333; c) J. A. Caram, E. Vasini, *J. Electrochim. Acta* **1994**, *39*, 2395.

[5] Y. Y. Lim, R. S. J. Drago, *Inorg. Chem.* **1972**, *11*, 1334.

[6] J. McMurry, *Organic Chemistry*, 4th ed., Brooks/Cole Publishing Co., New York, **1999**.

[7] U. Kreher, S. Schebesta, D. Walther, *Z. Anorg. Allg. Chem.* **1998**, *624*, 602.
